# Supplementary material for: Bundling and segregation affect pheromone deposition, but not choice, in an ant
Source: eLife. 2022 Nov 22;11:e79314. doi: 10.7554/eLife.79314 (PMC9681204; doi:10.7554/eLife.79314)
Supplement: Supplementary file 1. — Entire statistical analysis code produced with R and Python, including data handling, figure code, and analysis results. [file elife-79314-supp1.pdf]

Bundling and segregation affects pheromone deposition, but not choice, in an ant

ESM2 - Analysis

Massimo De Agrò, Chiara Matschunas, and Tomer J. Czaczkes

Intro

This supplement provides the entire R script and output of the statistical analysis we performed and figures produced, in their original form. It is presented in the spirit of open and transparent science, but has not been carefully curated.

Contents

Setup **2**
- Prepare R environment . . . 2
- Prepare Python environment . . . 2
- Load data . . . 2

Pilot Analysis **2**
- Binomial analysis . . . 2
- Pheromone analysis . . . 4
  - Graph . . . 7

Main experiment **8**
- Condition 1 - Segregated reward VS Bundled . . . 8
  - Binomial analysis . . . 8
  - Pheromone analysis . . . 10
- Condition 2 - Segregated reward VS Segregated All . . . 14
  - Binomial analysis . . . 14
  - Pheromone analysis . . . 16
- Condition 3 - Segregated All VS Bundled . . . 21
  - Binomial analysis . . . 21
  - Pheromone analysis . . . 23
- Overall pheromone Graph . . . 28

Further investigation **29**
- Is the difference between condition caused by contrast effect? . . . 29
  - Condition 1 . . . 29
  - Condition 2 . . . 31
  - Condition 3 . . . 33
  - Graph . . . 35
- No visit 1 . . . 38
  - Condition 1 . . . 38
  - Condition 2 . . . 40
  - Condition 3 . . . 41
  - Graph . . . 43

Setup

Prepare R environment

```
library(glmmTMB) #for mixed models
library(car) #for anova on mixed models
library(DHARMa) #for goodness of fit of the model
library(emmeans) #for post hoc
library(reticulate)
library(reshape2)
library(effectsize)
library(ggplot2)
```

Prepare Python environment

```
import pandas as pd #to load data
import matplotlib.pyplot as plt #to plot
import numpy as np
import seaborn as sns #to plot
```

Load data

I will load both on R and on python. Description for each column can be found in the spreadsheet file.

```
pilot <- read.csv(paste0(pathtofile,'pilot.csv'))
cond1 <- read.csv(paste0(pathtofile,'cond1.csv'))
cond2 <- read.csv(paste0(pathtofile,'cond2.csv'))
cond3 <- read.csv(paste0(pathtofile,'cond3.csv'))
```

```
pilot = pd.read_csv(pathtofile+'pilot.csv')
cond1 = pd.read_csv(pathtofile+'cond1.csv')
cond2 = pd.read_csv(pathtofile+'cond2.csv')
cond3 = pd.read_csv(pathtofile+'cond3.csv')
```

Pilot Analysis

Binomial analysis

We want to know if ants chose significantly more the short over the long runway associated odour. Concurrently, we need to evaluate the usefulness of the subsequent tests on the final results. The initial choice generally indicates the first instinct of the animal, while in the final choice the ant had more time to ponder and can reconsider. For the same reason we need to include also the subsequent test visits, as the absence of a reward could influence subsequent choices. All will be included in the analysis.

```
pilotLong <- melt(pilot, measure.vars = c("Initial_Choice_Short", "Final_Choice_Short"),
                  value.name = "choice", na.rm = TRUE, variable.name = "order")

mpm <- glmmTMB(choice~as.factor(Visit_Number)*order+(1|Colony/Ant_ID_Long),
               data = pilotLong, family = binomial)

#Check the goodness of fit
simres <- simulateResiduals(mpm)
plot(simres, factor=TRUE)
```

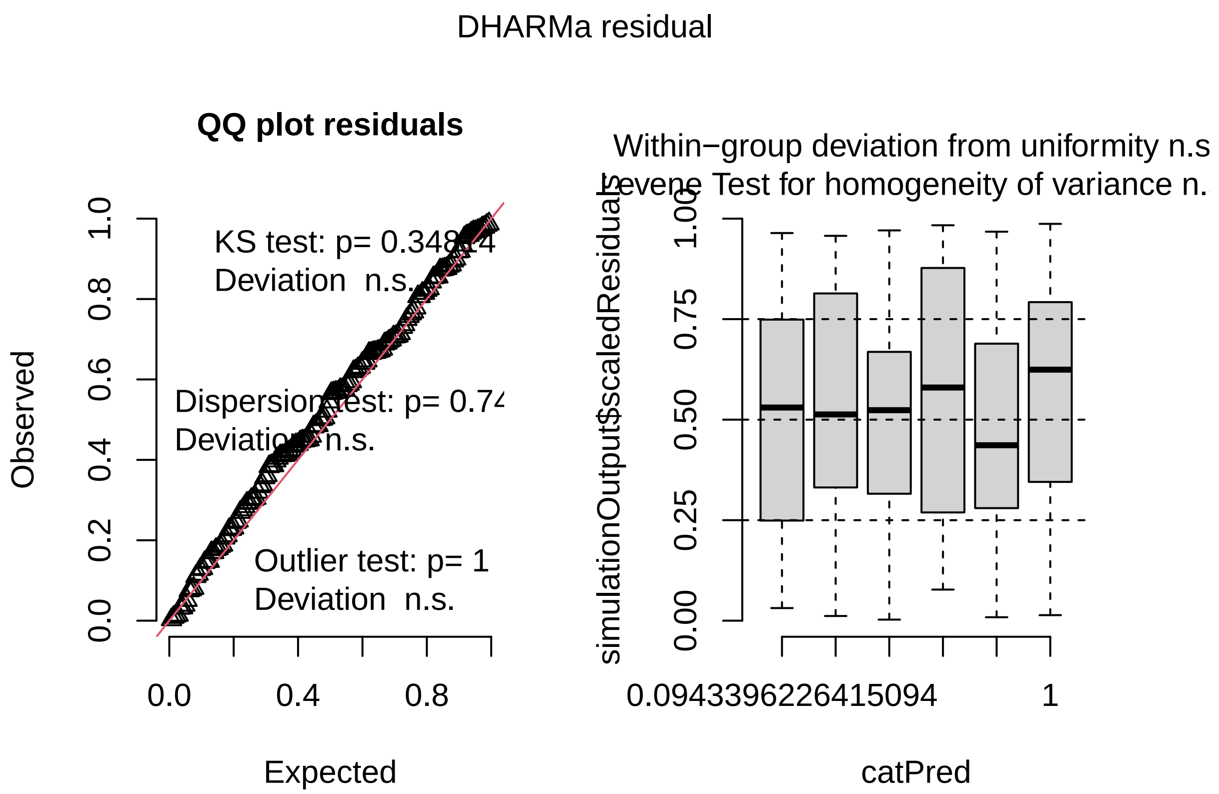

The data is distributed correctly, I proceed with analysis of deviance

```
Anova(mpm)
```

```
## Analysis of Deviance Table (Type II Wald chisquare tests)
##
## Response: choice
##                                  Chisq Df Pr(>Chisq)
## as.factor(Visit_Number)        13.8457  2   0.000985 ***
## order                           0.3528  1   0.552533
## as.factor(Visit_Number):order   2.8948  2   0.235180
## ---
## Signif. codes:  0 '***' 0.001 '**' 0.01 '*' 0.05 '.' 0.1 ' ' 1
```

Indeed there is a difference between subsequent testing visits. However, there is no difference between initial and final choices. I proceed with post-hoc on relevant variables.

```
e <- emmeans(mpm, ~Visit_Number, type='response')
```

```
## NOTE: Results may be misleading due to involvement in interactions
```

```
t<-test(e, adjust='bonferroni')
t
```

```
##  Visit_Number  prob    SE  df null t.ratio p.value
##             5 0.896 0.056 136  0.5   3.586  0.0014
##             6 0.547 0.124 136  0.5   0.376  1.0000
##             7 0.484 0.123 136  0.5  -0.129  1.0000
##
## Results are averaged over the levels of: order
## P value adjustment: bonferroni method for 3 tests
## Tests are performed on the logit scale
```

```
t$t.ratio
```

```
## [1]  3.5863276  0.3759206 -0.1287224
```

```
interpret_cohens_d(t_to_d(t = t$t.ratio, df = t$df))
```

```
## d    |       95% CI | Interpretation
## ------------------------------------
## 0.62 | [0.27, 0.96] |         medium
## 0.06 | [0.27, 0.40] |     very small
## 0.02 | [0.36, 0.31] |     very small
##
## - Interpretation rule: cohen1988
```

```
interpret_eta_squared(t_to_eta2(t = t$t.ratio, df = t$df))
```

```
## Eta2 (partial) |       95% CI | Interpretation
## ----------------------------------------------
## 0.09           | [0.03, 1.00] |         medium
## 1.04e-03       | [0.00, 1.00] |     very small
## 1.22e-04       | [0.00, 1.00] |     very small
##
## - One-sided CIs: upper bound fixed at [1.00].
## - Interpretation rule: field2013
```

the ants chose significantly more the short runways (89.6% of the times. The preference then quickly drop to chance level, as expected due to the missing reward.

This pilot results gives us evidence of the fact that indeed ants dislike walking more. We will also observe the pheromone deposition.

Pheromone analysis

a consideration: we expect the long runway to receive triple the pheromone (independently of other effects), just because it is three times long. For this reason, the pheromone will be adjusted by runway length. Rather than dividing by three the long one, I will multiply by 3 the short one, to be able to still use poisson distributions.

```
pilotPheroLong <- melt(pilot, measure.vars = c("Pheromone_Go", "Pheromone_Back"),
                  value.name = "Pheromone", na.rm = TRUE, variable.name = "direction")
pilotPheroLong$Pheromone_Adjusted <- pilotPheroLong$Pheromone
pilotPheroLong$Pheromone_Adjusted[pilotPheroLong$Visit_Length == 1] <-
  pilotPheroLong$Pheromone_Adjusted[pilotPheroLong$Visit_Length == 1]*3

mpp <- glmmTMB(Pheromone_Adjusted~direction*as.factor(Visit_Length)+(Visit_Number|Colony/Ant_ID_Long),
               data=pilotPheroLong, family = poisson())
simres <- simulateResiduals(mpp)
plot(simres, factor=TRUE)
```

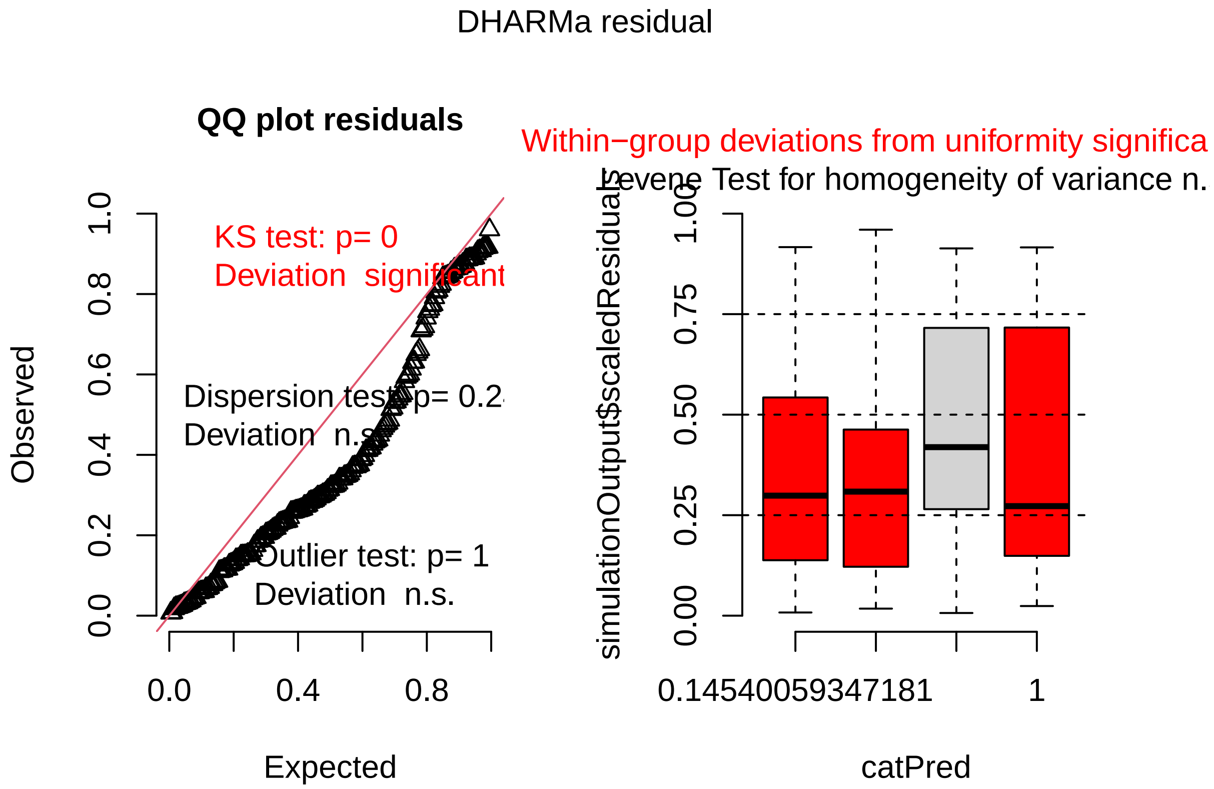

The distribution is not perfect. let's observe

```
hist(pilotPheroLong$Pheromone_Adjusted)
```

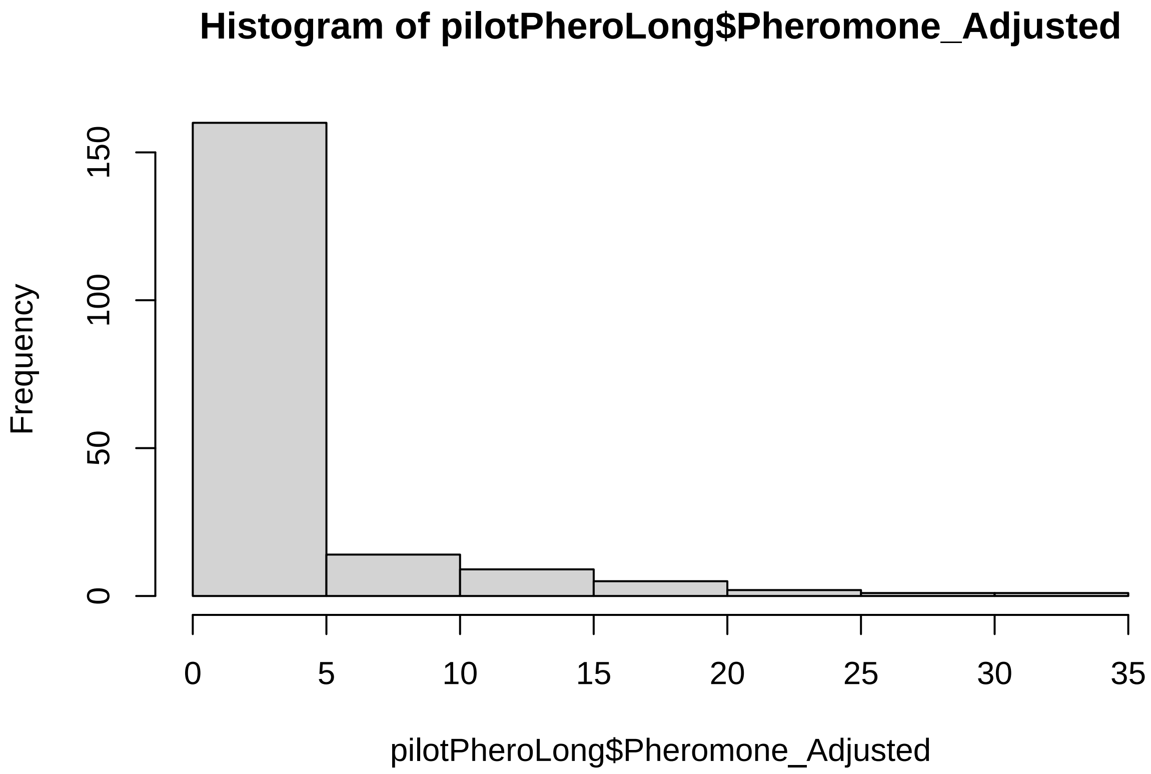

as expected lots of zeros. will add zero inflation control

```
mpp <- glmmTMB(Pheromone_Adjusted~direction*as.factor(Visit_Length)+(1|Colony/Ant_ID_Long),
               ziformula = ~1, data=pilotPheroLong, family = poisson())
#Adding Visit Number causes failure to converge. Not including it.
simres <- simulateResiduals(mpp)
plot(simres, factor=TRUE)
```

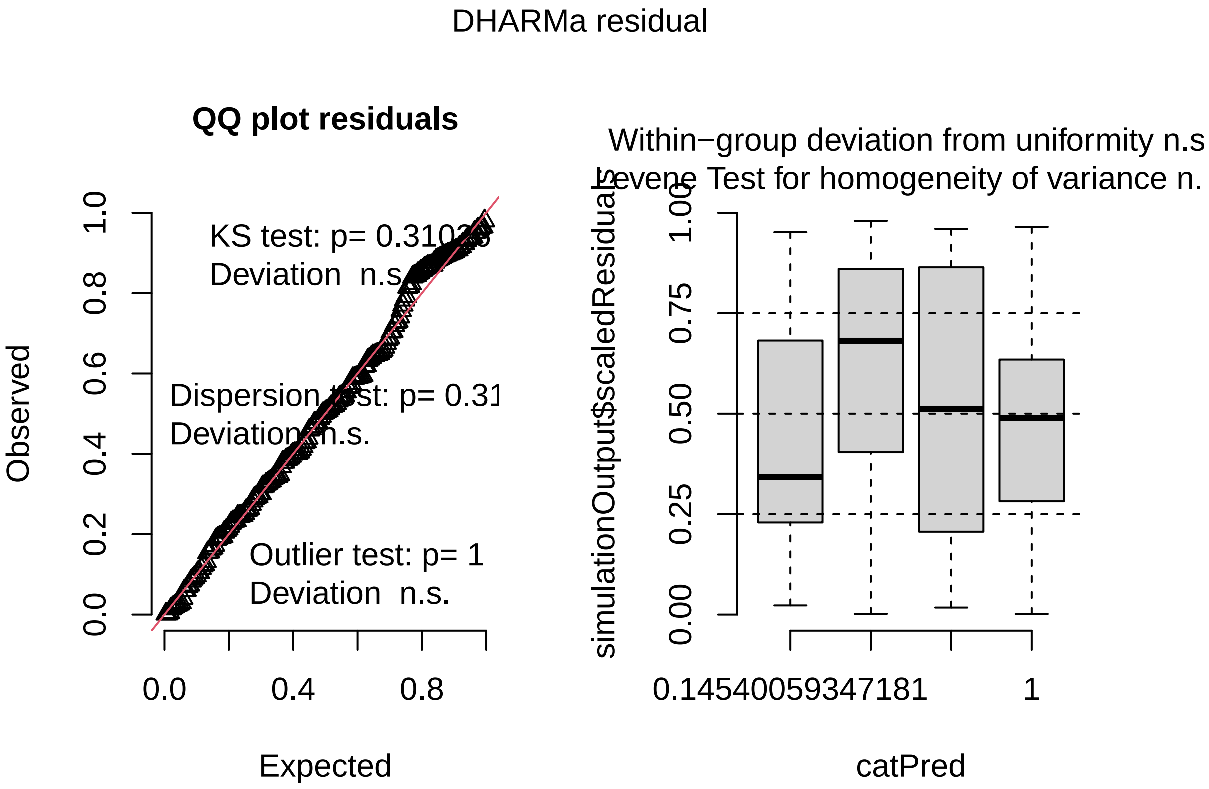

perfect. I proceed with analysis

```
Anova(mpp)
```

```
## Analysis of Deviance Table (Type II Wald chisquare tests)
##
## Response: Pheromone_Adjusted
##                                     Chisq Df Pr(>Chisq)
## direction                          0.0645  1     0.7995
## as.factor(Visit_Length)           34.7149  1  3.817e-09 ***
## direction:as.factor(Visit_Length)  2.3773  1     0.1231
## ---
## Signif. codes:  0 '***' 0.001 '**' 0.01 '*' 0.05 '.' 0.1 ' ' 1
```

there seems to be no difference between the way towards the drop or back. However there is a big difference between the long and short options.

```
e <- emmeans(mpp, ~Visit_Length, type='response')
```

```
## NOTE: Results may be misleading due to involvement in interactions
```

```
e
```

```
##  Visit_Length rate   SE  df lower.CL upper.CL
##             1 3.03 2.09 185    0.778    11.82
##             3 1.52 1.04 185    0.394     5.86
```

```
##
## Results are averaged over the levels of: direction
## Confidence level used: 0.95
## Intervals are back-transformed from the log scale
```

```
t <- pairs(e)
t
```

```
##  contrast ratio    SE  df null t.ratio p.value
##  1 / 3        2 0.231 185    1   5.973  <.0001
##
## Results are averaged over the levels of: direction
## Tests are performed on the log scale
```

```
t <- as.data.frame(t)

interpret_cohens_d(t_to_d(t = t$t.ratio, df = t$df))
```

```
## d    |       95% CI | Interpretation
## ------------------------------------
## 0.88 | [0.58, 1.18] |          large
##
## - Interpretation rule: cohen1988
```

```
interpret_eta_squared(t_to_eta2(t = t$t.ratio, df = t$df))
```

```
## Eta2 (partial) |       95% CI | Interpretation
## ----------------------------------------------
## 0.16           | [0.09, 1.00] |          large
##
## - One-sided CIs: upper bound fixed at [1.00].
## - Interpretation rule: field2013
```

The pheromone deposited is higher for the short runway. However, is important to interpret these result with caution, as the pheromone deposition may not linearly increase with length at all and so our adjustment may be improper.

Graph

first of all, I will prepare the post-hoc tables for plotting

```
pilotPheroToPlot <- as.data.frame(e)
```

```
pilotPhero = pd.DataFrame(r.pilotPheroToPlot)

fig, ax = plt.subplots()
ax.scatter(pilotPhero['Visit_Length'].values, pilotPhero['rate'].values)
ax.vlines(pilotPhero['Visit_Length'].values,
          ymin=np.subtract(pilotPhero['rate'].values,pilotPhero['SE'].values),
          ymax=np.add(pilotPhero['rate'].values,pilotPhero['SE'].values))
```

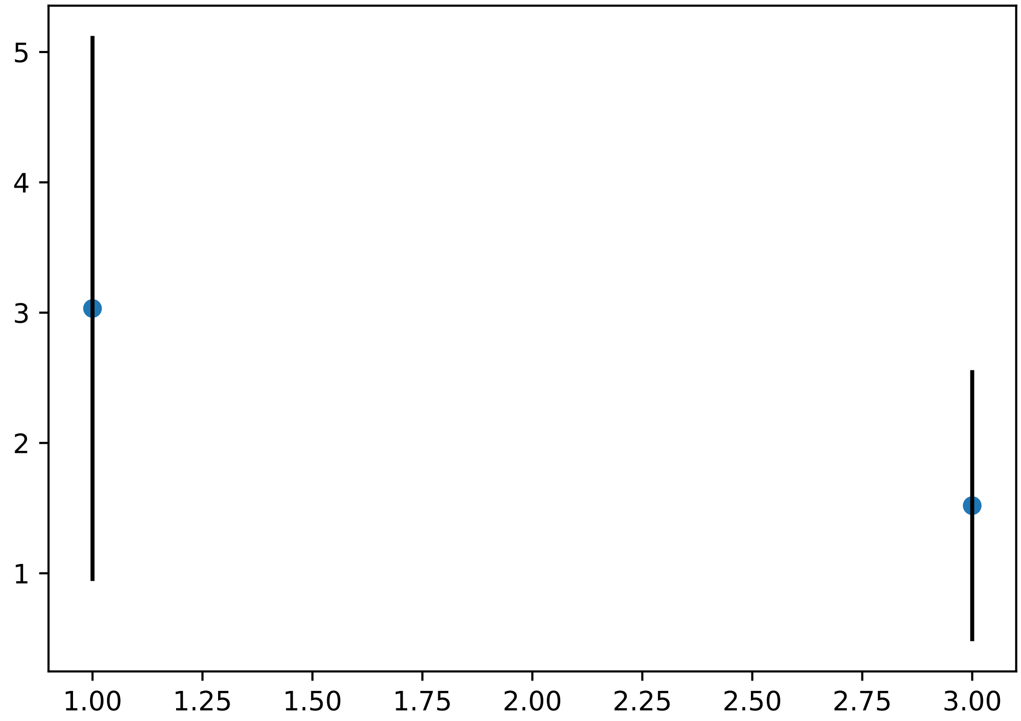

Main experiment

We will follow the exact same analysis for all three conditions of the main experiment, as we did for the pilot. Namely, we will look at the binomial decision first, and at the pheromone deposition later.

Condition 1 - Segregated reward VS Bundled

Binomial analysis

```
cond1Long <- melt(cond1, measure.vars = c("Initial_Choice_Binomial", "Final_Choice_Binomial"),
                  value.name = "choice", na.rm = TRUE, variable.name = "order")

mc1m <- glmmTMB(choice~as.factor(Visit_number)*order+(1|Colony/Ant_ID_long),
                data = cond1Long, family = binomial,
                control=glmmTMBControl(optCtrl = list(iter.max = 300000, eval.max = 400000)))

#Check the goodness of fit
simres <- simulateResiduals(mc1m)
plot(simres, factor=TRUE)
```

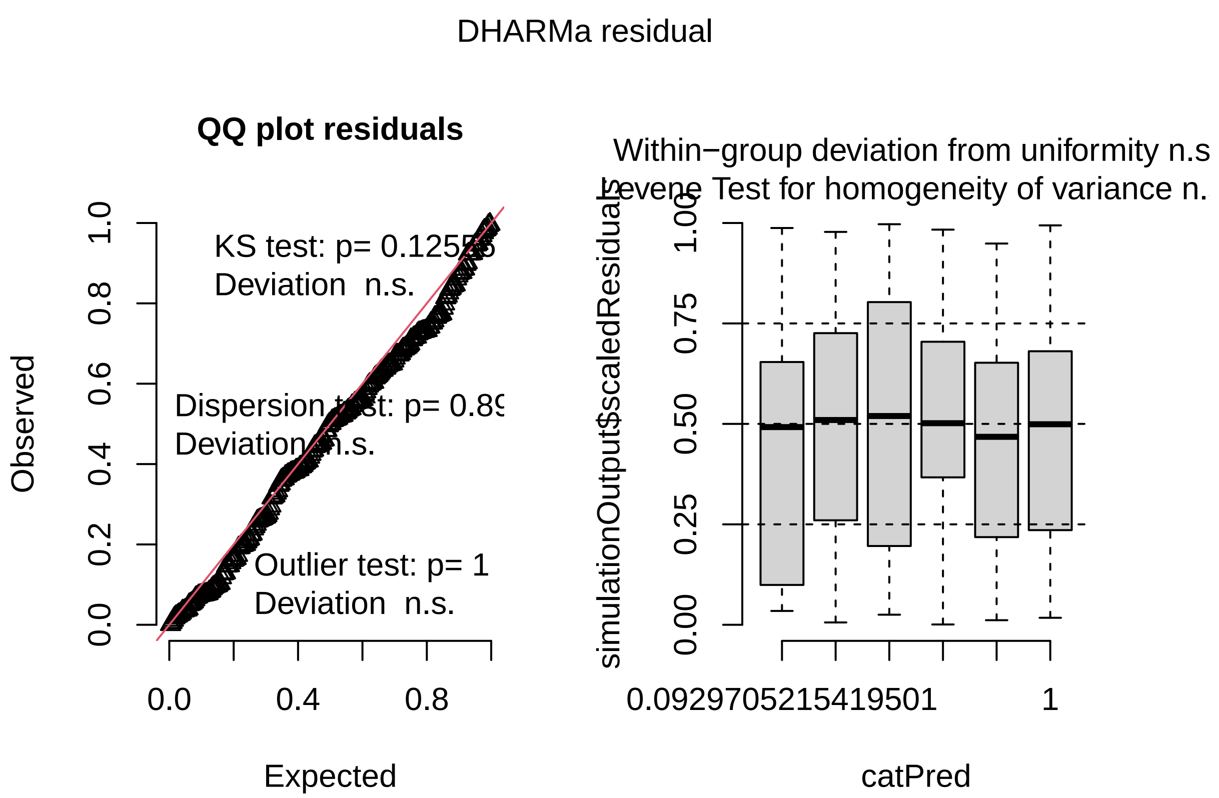


```
Anova(mc1m)
```

```
## Analysis of Deviance Table (Type II Wald chisquare tests)
##
## Response: choice
##                                 Chisq Df Pr(>Chisq)
## as.factor(Visit_number)        9.5744  2   0.008336 **
## order                          0.0813  1   0.775590
## as.factor(Visit_number):order 0.3063  2   0.858003
## ---
## Signif. codes:  0 '***' 0.001 '**' 0.01 '*' 0.05 '.' 0.1 ' ' 1
```

there is an effect of visit number, but not of initial vs final choice

```
e <- emmeans(mc1m, ~Visit_number, type='response')
```

```
## NOTE: Results may be misleading due to involvement in interactions
```

```
t<-test(e, adjust='bonferroni')
t
```

```
##  Visit_number  prob     SE  df null t.ratio p.value
##             9 0.516 0.0693 232  0.5   0.227  1.0000
##            10 0.729 0.0596 232  0.5   3.279  0.0036
##            11 0.486 0.0693 232  0.5  -0.195  1.0000
##
## Results are averaged over the levels of: order
## P value adjustment: bonferroni method for 3 tests
## Tests are performed on the logit scale
```

```
interpret_cohens_d(t_to_d(t = t$t.ratio, df = t$df))
```

```
## d    |       95% CI | Interpretation
## ------------------------------------
## 0.03 | [0.23, 0.29] |     very small
## 0.43 | [0.17, 0.69] |          small
## 0.03 | [0.28, 0.23] |     very small
##
## - Interpretation rule: cohen1988
```

```
interpret_eta_squared(t_to_eta2(t = t$t.ratio, df = t$df))
```

```
## Eta2 (partial) |       95% CI | Interpretation
## ----------------------------------------------
## 2.23e-04       | [0.00, 1.00] |     very small
## 0.04           | [0.01, 1.00] |          small
## 1.64e-04       | [0.00, 1.00] |     very small
##
## - One-sided CIs: upper bound fixed at [1.00].
## - Interpretation rule: field2013
```

```
#saving for later
cond1ToPlot <- as.data.frame(e)
e <- emmeans(mc1m, ~Visit_number*order, type='response')
test(e)
```

```
##  Visit_number order                   prob     SE  df null t.ratio p.value
##             9 Initial_Choice_Binomial 0.530 0.0917 232  0.5   0.330  0.7415
##            10 Initial_Choice_Binomial 0.701 0.0818 232  0.5   2.186  0.0298
##            11 Initial_Choice_Binomial 0.472 0.0918 232  0.5  -0.306  0.7599
##             9 Final_Choice_Binomial   0.501 0.0920 232  0.5   0.012  0.9902
##            10 Final_Choice_Binomial   0.755 0.0754 232  0.5   2.762  0.0062
##            11 Final_Choice_Binomial   0.501 0.0920 232  0.5   0.012  0.9902
##
## Tests are performed on the logit scale
```

```
cond1ToPlotDetailed <- as.data.frame(e)
```

there is a significant preference for the segregated option. Strangely enough however, it's only for the second test, while for the first and the last the choice remains random.

Pheromone analysis

Here the two options are equally long, so no need for adjusting. however, they are fundamentally divided into three steps. As such we will analyze the pheromone deposited as three separate steps. We did not record the pheromone deposited the way to the drops, as it was impossible to keep track of it while dispensing the small drops.

```
cond1PheroLong <- melt(cond1, measure.vars = c("Pherom_back_1", "Pherom_back_2","Pherom_back_3"),
                  value.name = "Pheromone", na.rm = TRUE, variable.name = "section")

mc1p <- glmmTMB(Pheromone~section*Treatment+(1|Visit_number/Colony/Ant_ID_long),
                data=cond1PheroLong, family = poisson())
simres <- simulateResiduals(mc1p)
plot(simres, factor=TRUE)
```

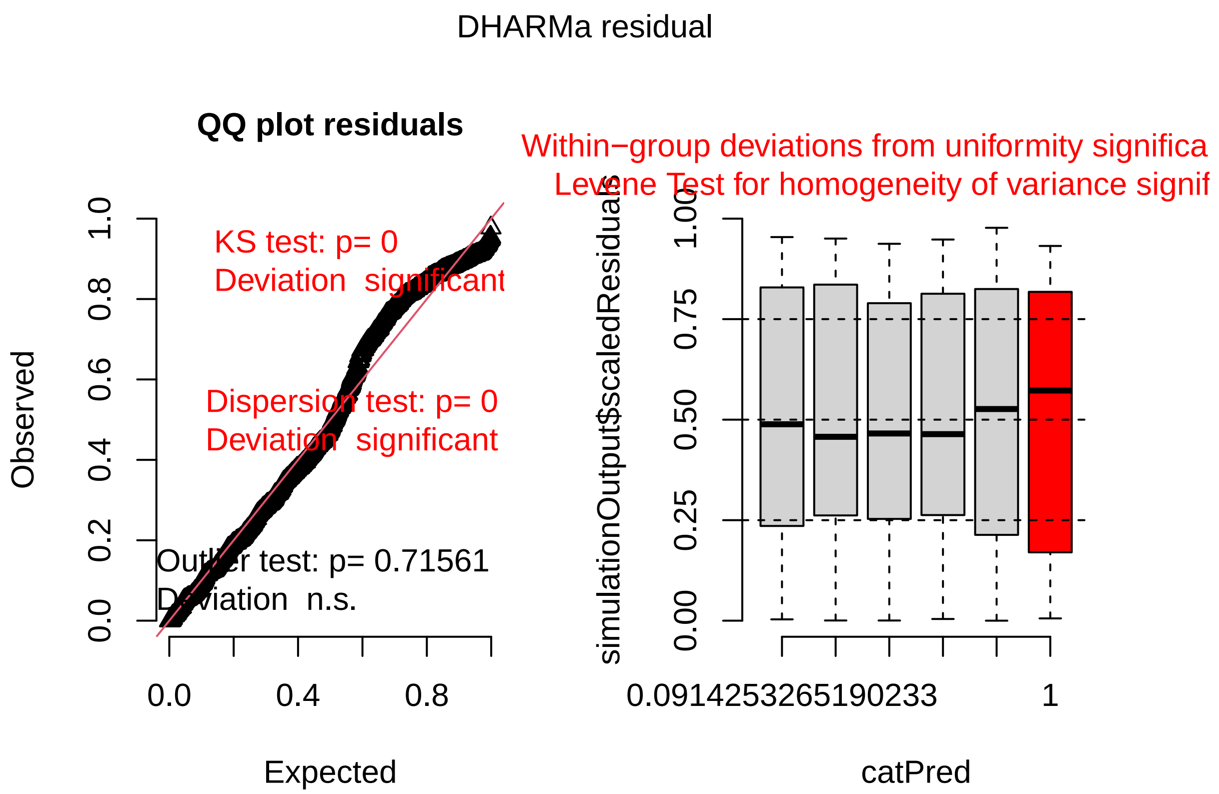

model is not good. probably zero inflated. Will check

```
hist(cond1PheroLong$Pheromone)
```

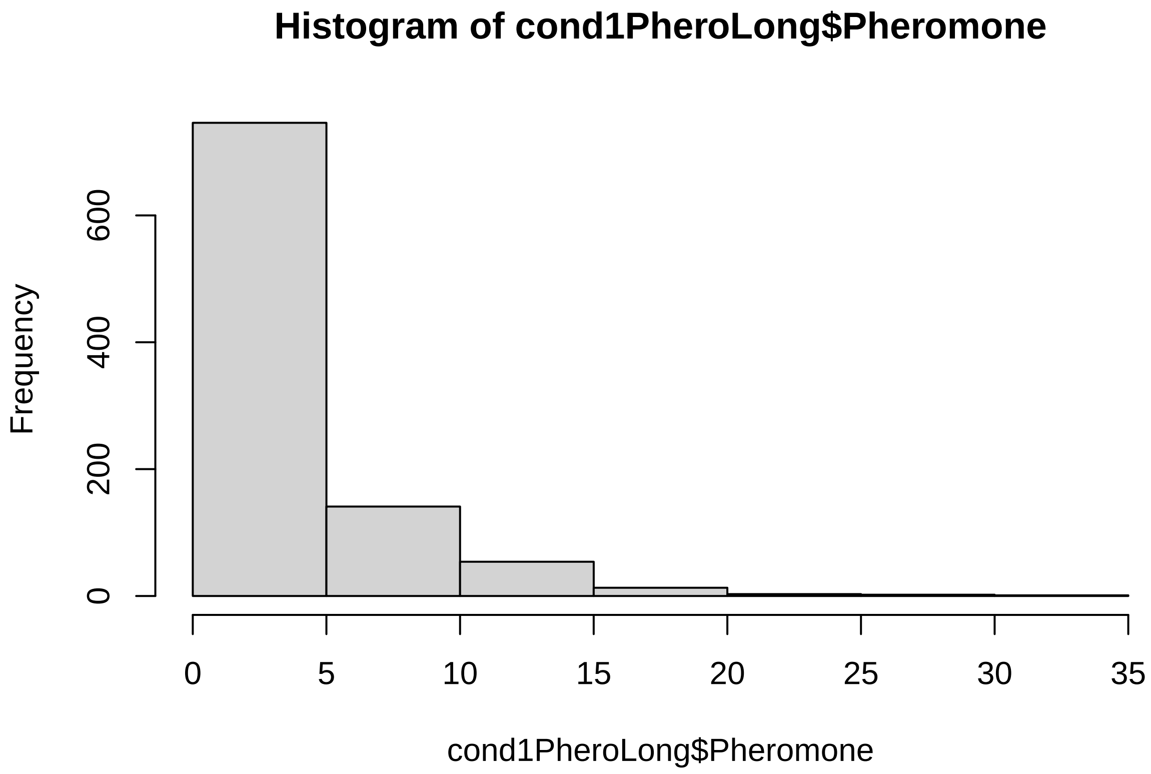

```r
#indeed zero inflated
mc1p <- glmmTMB(Pheromone~section*Treatment+(1|Colony/Ant_ID_long), data=cond1PheroLong,
                ziformula = ~1, family = poisson())
simres <- simulateResiduals(mc1p)
plot(simres, factor=TRUE)
```

```
## DHARMa:testOutliers with type = binomial may have inflated Type I error rates for integer-valued dis
```

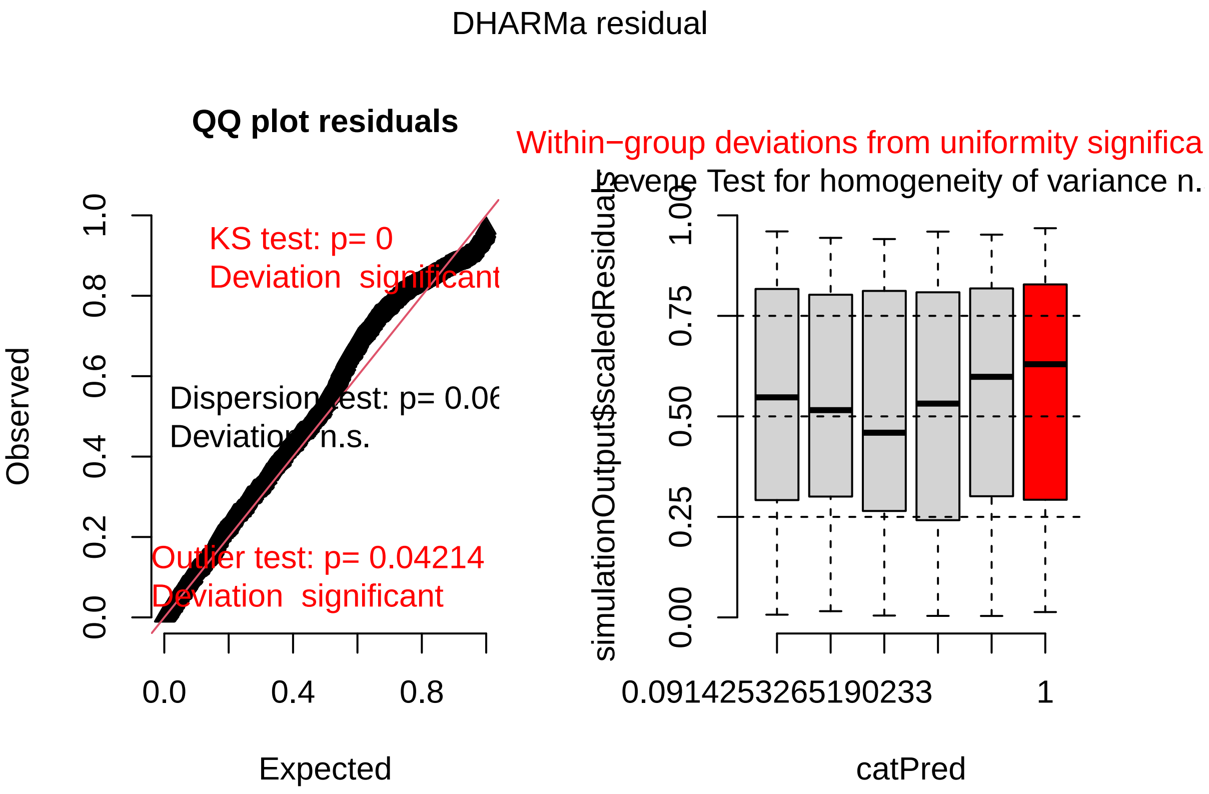

not working. will try tweedie again?

```r
mc1p <- glmmTMB(Pheromone~section*Treatment+(1|Visit_number/Colony/Ant_ID_long),
                ziformula = ~1, data=cond1PheroLong, family = tweedie(link = 'log'))
simres <- simulateResiduals(mc1p)
plot(simres, factor=TRUE)
```

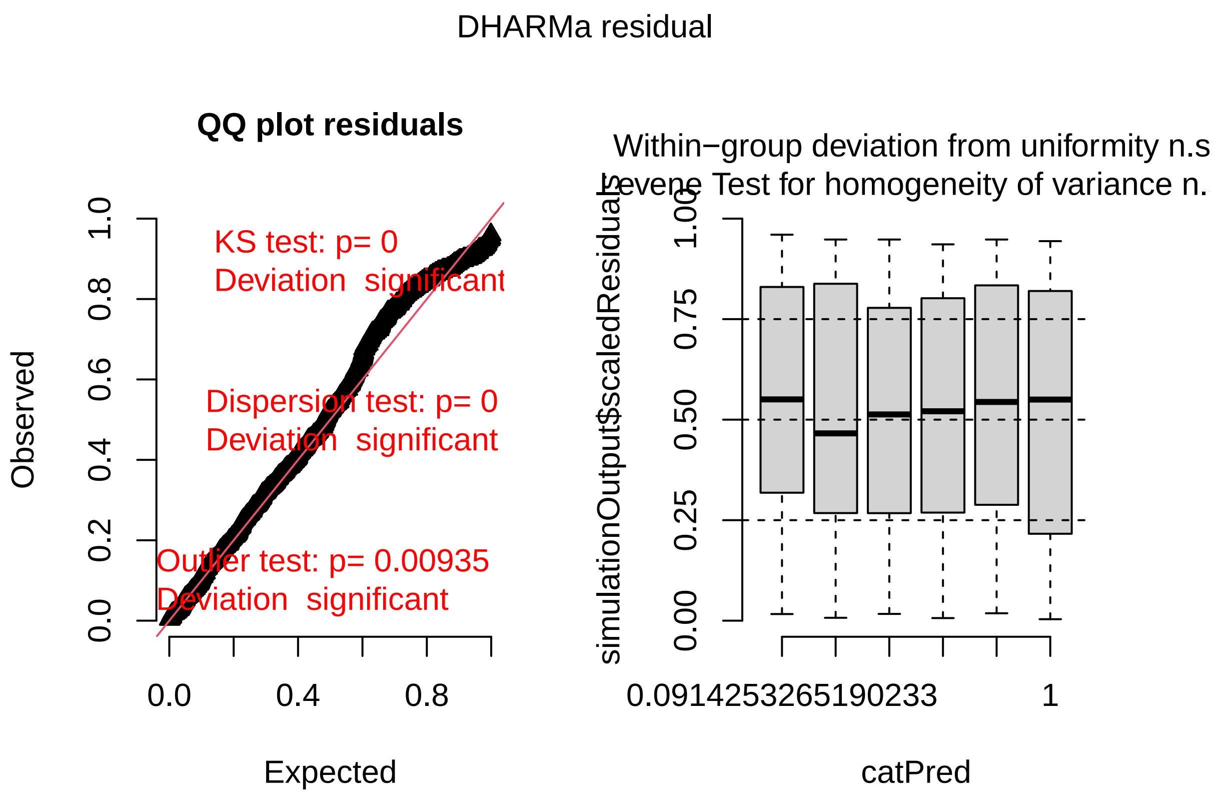

I don't think is getting better than this. will proceed.

```
Anova(mc1p)
```

```
## Analysis of Deviance Table (Type II Wald chisquare tests)
##
## Response: Pheromone
##                      Chisq Df Pr(>Chisq)
## section           107.2664  2     <2e-16 ***
## Treatment           2.0487  1     0.1523
## section:Treatment   0.6412  2     0.7257
## ---
## Signif. codes:  0 '***' 0.001 '**' 0.01 '*' 0.05 '.' 0.1 ' ' 1
```

there is a strong effect, but no interaction. will proceed

```
e <- emmeans(mc1p, ~Treatment*section, type="response")
e
```

```
##  Treatment         section       response    SE  df lower.CL upper.CL
##  Bundled           Pherom_back_1    0.878 0.200 948    0.561    1.374
##  Reward_Segregated Pherom_back_1    1.276 0.278 948    0.831    1.957
##  Bundled           Pherom_back_2    0.566 0.131 948    0.359    0.892
##  Reward_Segregated Pherom_back_2    0.823 0.182 948    0.533    1.270
##  Bundled           Pherom_back_3    0.495 0.115 948    0.313    0.782
##  Reward_Segregated Pherom_back_3    0.653 0.146 948    0.421    1.013
##
## Confidence level used: 0.95
## Intervals are back-transformed from the log scale
```

```r
t <- contrast(e, list( "bundledVsSegregatedReward" = c(1/3,-1/3,1/3,-1/3,1/3,-1/3),
                  "Section1VsSection2" = c(0.5,0.5,-0.5,-0.5,0,0),
                  "Section2VsSection3" = c(0,0,0.5,0.5,-0.5,-0.5),
                  "Section1VsSection3" = c(0.5,0.5,0,0,-0.5,-0.5)), adjust = "bonferroni")

t
```

```
##  contrast                  ratio     SE  df null t.ratio p.value
##  bundledVsSegregatedReward  0.71 0.1735 948    1  -1.399  0.6482
##  Section1VsSection2         1.55 0.0955 948    1   7.124  <.0001
##  Section2VsSection3         1.20 0.0842 948    1   2.599  0.0380
##  Section1VsSection3         1.86 0.1205 948    1   9.598  <.0001
##
## P value adjustment: bonferroni method for 4 tests
## Tests are performed on the log scale
```

```r
t<-as.data.frame(t)
interpret_cohens_d(t_to_d(t = t$t.ratio, df = t$df))
```

```
## d    |       95% CI | Interpretation
## ------------------------------------
## 0.09 | [0.22, 0.04] |     very small
## 0.46 | [0.33, 0.59] |          small
## 0.17 | [0.04, 0.30] |     very small
## 0.62 | [0.49, 0.75] |         medium
##
## - Interpretation rule: cohen1988
```

```r
interpret_eta_squared(t_to_eta2(t = t$t.ratio, df = t$df))
```

```
## Eta2 (partial) |       95% CI | Interpretation
## ----------------------------------------------
## 2.06e-03       | [0.00, 1.00] |     very small
## 0.05           | [0.03, 1.00] |          small
## 7.08e-03       | [0.00, 1.00] |     very small
## 0.09           | [0.06, 1.00] |         medium
##
## - One-sided CIs: upper bound fixed at [1.00].
## - Interpretation rule: field2013
```

```r
etoplot <- emmeans(mc1p, ~Treatment, type="response")
```

```
## NOTE: Results may be misleading due to involvement in interactions
```

```r
exp1PheroToplot <- as.data.frame(etoplot)
write.csv(exp1PheroToplot,paste0(pathtofile, 'cond1Phero.csv'))
```

We find an overall increased deposition for the section nearest the drop. There is an almost significant increased deposition for the segregated drop, but the p-value is just shy of 0.05.

Condition 2 - Segregated reward VS Segregated All

Binomial analysis

```r
cond2Long <- melt(cond2, measure.vars = c("Initial_Choice_Binomial", "Final_Choice_Binomial"),
                  value.name = "choice", na.rm = TRUE, variable.name = "order")
```

```r
mc2m <- glmmTMB(choice~as.factor(Visit_number)*order+(1|Colony/Ant_ID_long),
                data = cond2Long, family = binomial,
                control=glmmTMBControl(optCtrl = list(iter.max = 300000, eval.max = 400000)))

#Check the goodness of fit
simres <- simulateResiduals(mc2m)
plot(simres, factor=TRUE)
```

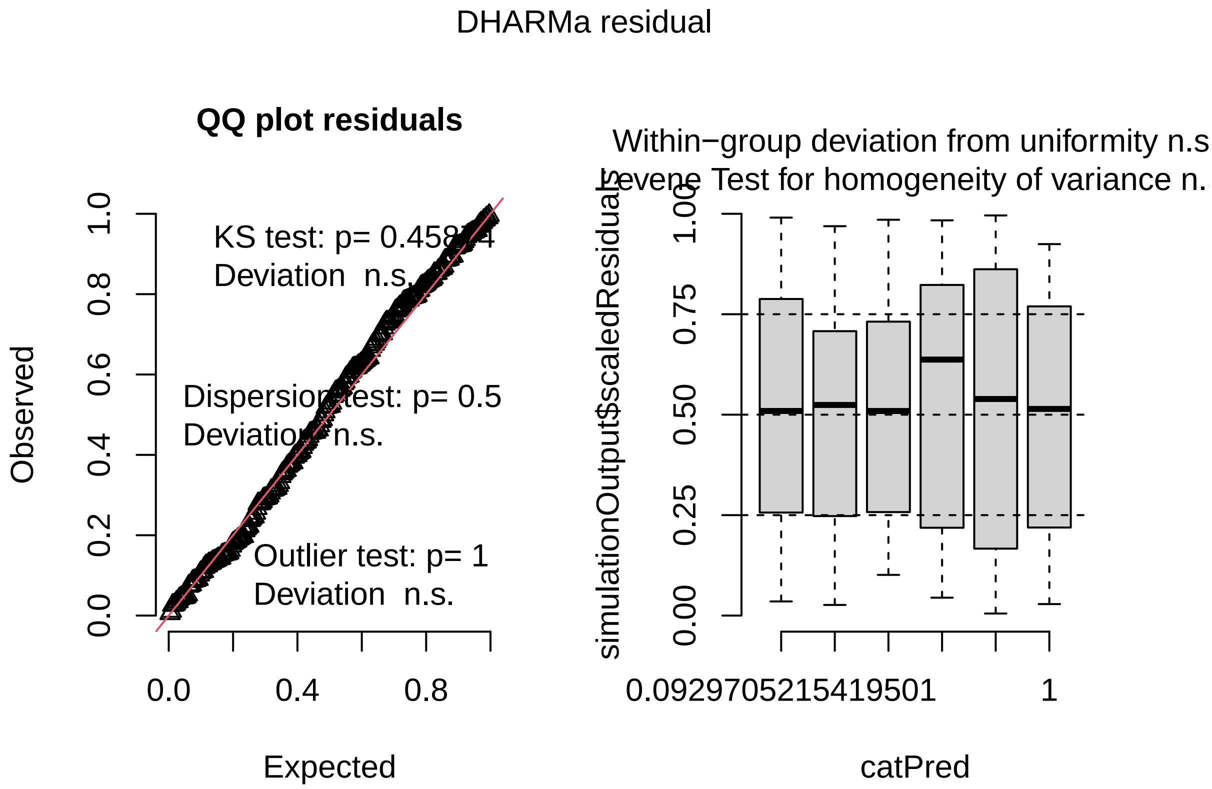


```r
Anova(mc2m)
```

```
## Analysis of Deviance Table (Type II Wald chisquare tests)
##
## Response: choice
##                                  Chisq Df Pr(>Chisq)
## as.factor(Visit_number)         1.5784  2     0.4542
## order                           0.1951  1     0.6587
## as.factor(Visit_number):order 0.3905  2     0.8226
```

no effect

```r
e <- emmeans(mc2m, ~1, type='response')
t<-test(e, adjust='bonferroni')
t
```

```
##  1        prob   SE  df null t.ratio p.value
##  overall 0.524 0.06 232  0.5   0.402  0.6880
##
## Results are averaged over the levels of: Visit_number, order
## Tests are performed on the logit scale
```

```r
t<-as.data.frame(t)
interpret_cohens_d(t_to_d(t = t$t.ratio, df = t$df))
```

```
## d    |       95% CI | Interpretation
## ------------------------------------
## 0.05 | [0.20, 0.31] |     very small
##
## - Interpretation rule: cohen1988
```

```r
interpret_eta_squared(t_to_eta2(t = t$t.ratio, df = t$df))
```

```
## Eta2 (partial) |       95% CI | Interpretation
## ----------------------------------------------
## 6.96e-04       | [0.00, 1.00] |     very small
##
## - One-sided CIs: upper bound fixed at [1.00].
## - Interpretation rule: field2013
```

and no real preference. Will check divided by visit for completeness.

```r
e <- emmeans(mc2m, ~Visit_number*order, type='response')
test(e, adjust='bonferroni')
```

```
##  Visit_number order                    prob     SE  df null t.ratio p.value
##             9 Initial_Choice_Binomial 0.497 0.1019 232  0.5  -0.031  1.0000
##            10 Initial_Choice_Binomial 0.594 0.0992 232  0.5   0.926  1.0000
##            11 Initial_Choice_Binomial 0.432 0.1005 232  0.5  -0.668  1.0000
##             9 Final_Choice_Binomial   0.562 0.1008 232  0.5   0.608  1.0000
##            10 Final_Choice_Binomial   0.562 0.1008 232  0.5   0.608  1.0000
##            11 Final_Choice_Binomial   0.497 0.1019 232  0.5  -0.031  1.0000
##
## P value adjustment: bonferroni method for 6 tests
## Tests are performed on the logit scale
```

```r
#saving for later
cond2ToPlotDetailed <- as.data.frame(e)
e <- emmeans(mc2m, ~Visit_number*order, type='response')
cond2ToPlot <- as.data.frame(e)
```

looks very flat, with a slight preference for the segregated reward option

Pheromone analysis

```r
cond2PheroLong <- melt(cond2, measure.vars = c("Pherom_back_1", "Pherom_back_2","Pherom_back_3"),
                 value.name = "Pheromone", na.rm = TRUE, variable.name = "section")

mc2p <- glmmTMB(Pheromone~section*Treatment+(Visit_number|Colony/Ant_ID_long),
                data=cond2PheroLong, family = poisson())
simres <- simulateResiduals(mc2p)
plot(simres, factor=TRUE)
```

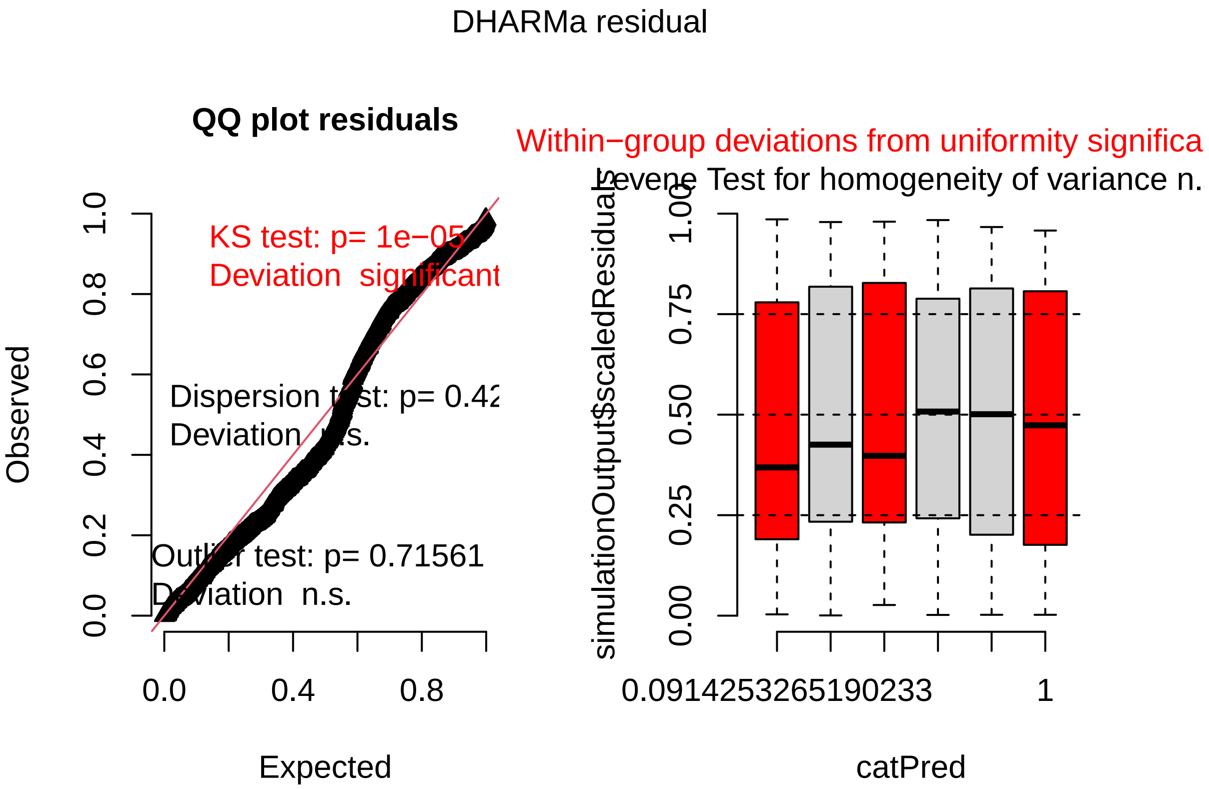

model is not good. probably zero inflated. Will check

```
hist(cond2PheroLong$Pheromone)
```

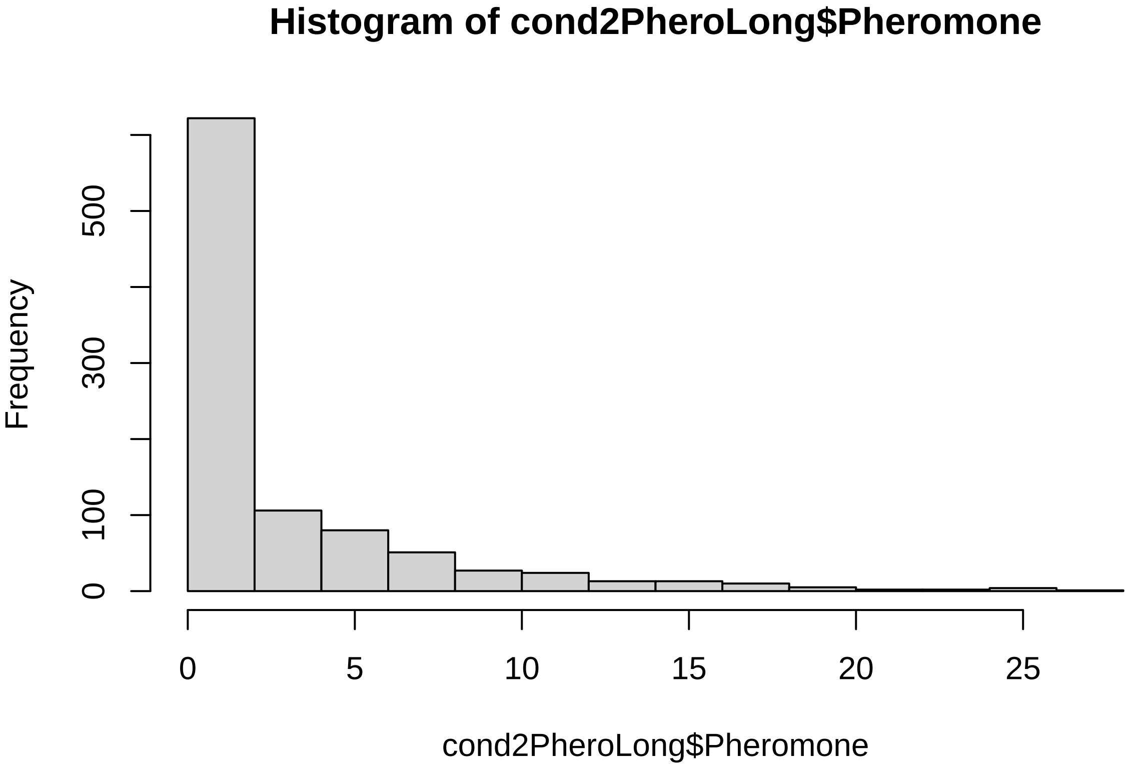

```r
#indeed zero inflated
mc2p <- glmmTMB(Pheromone~section*Treatment+(1|Visit_number/Colony/Ant_ID_long),
                data=cond2PheroLong, ziformula = ~1, family = poisson())
simres <- simulateResiduals(mc2p)
plot(simres, factor=TRUE)
```

```
## DHARMa:testOutliers with type = binomial may have inflated Type I error rates for integer-valued dis
```

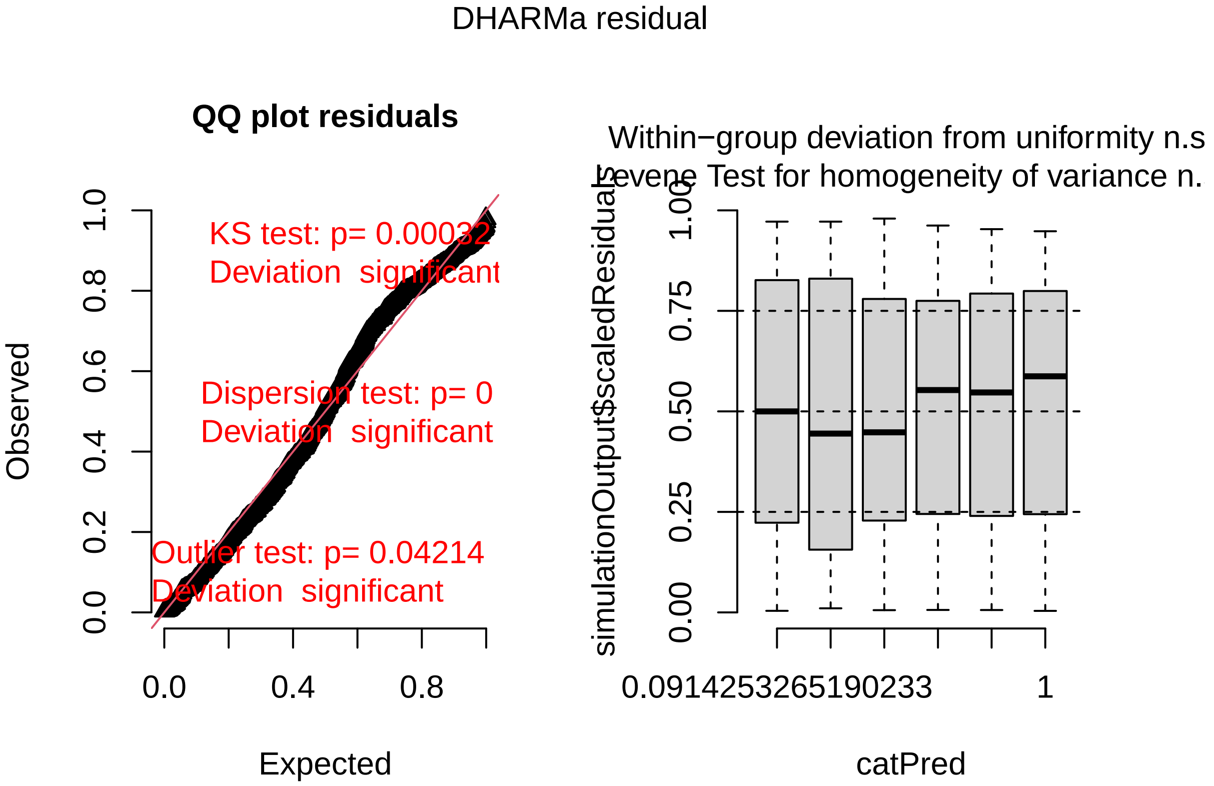

not perfect. will try tweedie again?

```r
mc2p <- glmmTMB(Pheromone~section*Treatment+(Visit_number|Colony/Ant_ID_long), ziformula = ~1,
                data=cond2PheroLong, family = tweedie(link = 'log'))
simres <- simulateResiduals(mc2p)
plot(simres, factor=TRUE)
```

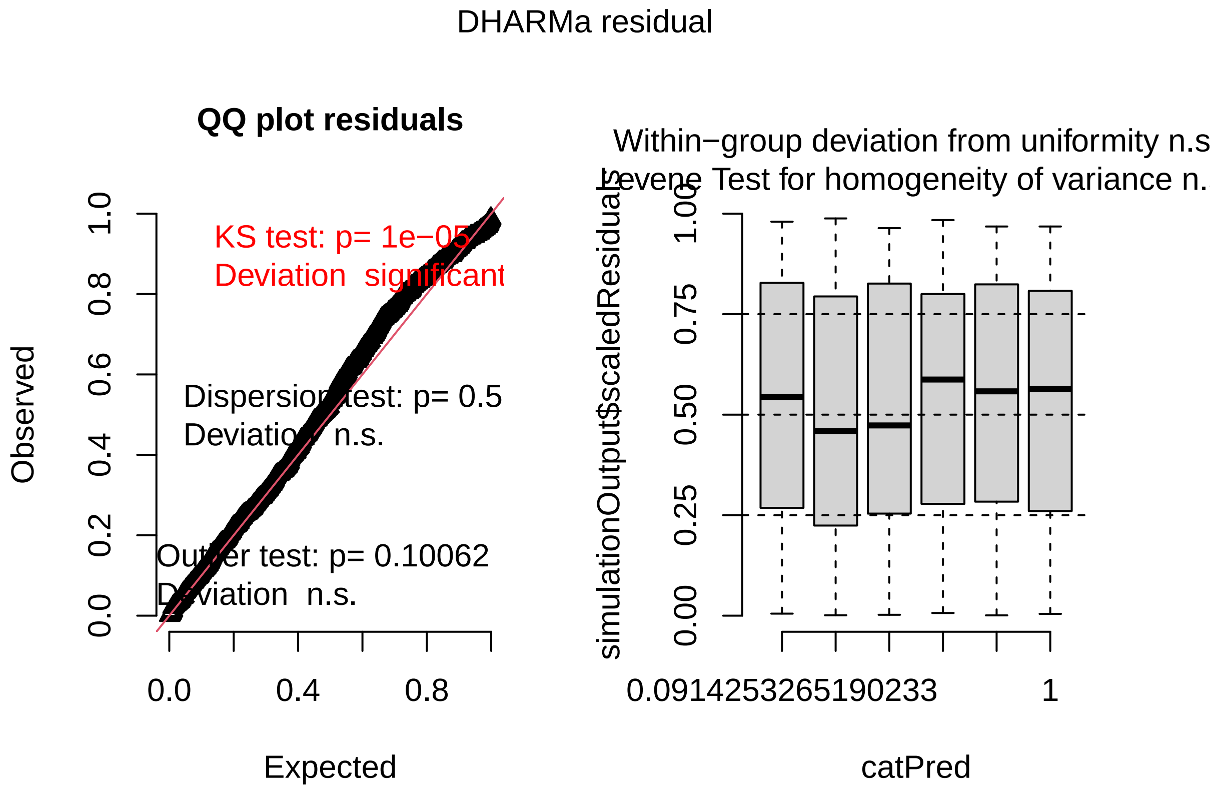

will proceed.

```
Anova(mc2p)
```

```
## Analysis of Deviance Table (Type II Wald chisquare tests)
##
## Response: Pheromone
##                     Chisq Df Pr(>Chisq)
## section           10.4118  2   0.005484 **
## Treatment         60.7675  1  6.423e-15 ***
## section:Treatment  5.6669  2   0.058811 .
## ---
## Signif. codes:  0 '***' 0.001 '**' 0.01 '*' 0.05 '.' 0.1 ' ' 1
```

there is again a strong effect, with almost significant interaction. will proceed

```
e <- emmeans(mc2p, ~Treatment*section, type="response")
e
```

```
##  Treatment          section       response    SE  df lower.CL upper.CL
##  All_Segregated     Pherom_back_1    0.835 0.306 945    0.407     1.71
##  Reward_Segregated Pherom_back_1    1.874 0.672 945    0.928     3.79
##  All_Segregated     Pherom_back_2    0.860 0.314 945    0.420     1.76
##  Reward_Segregated Pherom_back_2    1.549 0.560 945    0.762     3.15
##  All_Segregated     Pherom_back_3    0.819 0.298 945    0.401     1.67
##  Reward_Segregated Pherom_back_3    1.186 0.430 945    0.583     2.42
##
## Confidence level used: 0.95
## Intervals are back-transformed from the log scale
```

```r
t <- contrast(e, list( "SegregatedAllVsSegregatedReward" = c(1/3,-1/3,1/3,-1/3,1/3,-1/3),
                   "Section1VsSection2" = c(0.5,0.5,-0.5,-0.5,0,0),
                   "Section2VsSection3" = c(0,0,0.5,0.5,-0.5,-0.5),
                   "Section1VsSection3" = c(0.5,0.5,0,0,-0.5,-0.5),
                   "SegregatedAllSection1vs2"=c(1,0,-1,0,0,0),
                   "SegregatedAllSection2vs3"=c(0,0,1,0,-1,0),
                   "SegregatedAllSection1vs3"=c(1,0,0,0,-1,0),
                   "segregatedRewardSection1vs2"=c(0,1,0,-1,0,0),
                   "segregatedRewardSection2vs3"=c(0,0,0,1,0,-1),
                   "segregatedRewardSection1vs3"=c(0,1,0,0,0,-1)), adjust = "bonferroni")

t
```

```
##  contrast                        ratio     SE  df null t.ratio p.value
##  SegregatedAllVsSegregatedReward 0.555 0.0422 945    1  -7.746  <.0001
##  Section1VsSection2              1.084 0.0970 945    1   0.906  1.0000
##  Section2VsSection3              1.171 0.1078 945    1   1.712  0.8724
##  Section1VsSection3              1.269 0.1170 945    1   2.588  0.0980
##  SegregatedAllSection1vs2        0.972 0.1384 945    1  -0.200  1.0000
##  SegregatedAllSection2vs3        1.049 0.1470 945    1   0.344  1.0000
##  SegregatedAllSection1vs3        1.020 0.1471 945    1   0.137  1.0000
##  segregatedRewardSection1vs2     1.210 0.1316 945    1   1.752  0.8006
##  segregatedRewardSection2vs3     1.306 0.1554 945    1   2.243  0.2511
##  segregatedRewardSection1vs3     1.580 0.1809 945    1   3.995  0.0007
##
## P value adjustment: bonferroni method for 10 tests
## Tests are performed on the log scale
```

```r
t<-as.data.frame(t)
interpret_cohens_d(t_to_d(t = t$t.ratio, df = t$df))
```

```
## d        |       95% CI | Interpretation
## ----------------------------------------
## 0.50     | [0.63, 0.37] |         medium
## 0.06     | [0.07, 0.19] |     very small
## 0.11     | [0.02, 0.24] |     very small
## 0.17     | [0.04, 0.30] |     very small
## 0.01     | [0.14, 0.11] |     very small
## 0.02     | [0.11, 0.15] |     very small
## 8.89e-03 | [0.12, 0.14] |     very small
## 0.11     | [0.01, 0.24] |     very small
## 0.15     | [0.02, 0.27] |     very small
## 0.26     | [0.13, 0.39] |          small
##
## - Interpretation rule: cohen1988
```

```r
interpret_eta_squared(t_to_eta2(t = t$t.ratio, df = t$df))
```

```
## Eta2 (partial) |       95% CI | Interpretation
## ----------------------------------------------
## 0.06           | [0.04, 1.00] |          small
## 8.68e-04       | [0.00, 1.00] |     very small
## 3.09e-03       | [0.00, 1.00] |     very small
## 7.04e-03       | [0.00, 1.00] |     very small
## 4.24e-05       | [0.00, 1.00] |     very small
```

```
## 1.25e-04       | [0.00, 1.00] |     very small
## 1.97e-05       | [0.00, 1.00] |     very small
## 3.24e-03       | [0.00, 1.00] |     very small
## 5.30e-03       | [0.00, 1.00] |     very small
## 0.02           | [0.01, 1.00] |          small
##
## - One-sided CIs: upper bound fixed at [1.00].
## - Interpretation rule: field2013
```

```
etoplot <- emmeans(mc2p, ~Treatment, type="response")
```

```
## NOTE: Results may be misleading due to involvement in interactions
```

```
exp2PheroToplot <- as.data.frame(etoplot)
write.csv(exp2PheroToplot,paste0(pathtofile, 'cond2Phero.csv'))
```

Overall, more pheromone is deposited for the Segregated Reward over the Segregated Cost

Condition 3 - Segregated All VS Bundled

Binomial analysis

```
cond3Long <- melt(cond3, measure.vars = c("Initial_Choice_Binomial", "Final_Choice_Binomial"),
                  value.name = "choice", na.rm = TRUE, variable.name = "order")

mc3m <- glmmTMB(choice~as.factor(Visit_number)*order+(1|Colony/Ant_ID_long),
                data = cond3Long, family = binomial,
                control=glmmTMBControl(optCtrl = list(iter.max = 300000, eval.max = 400000)))

#Check the goodness of fit
simres <- simulateResiduals(mc3m)
plot(simres, factor=TRUE)
```

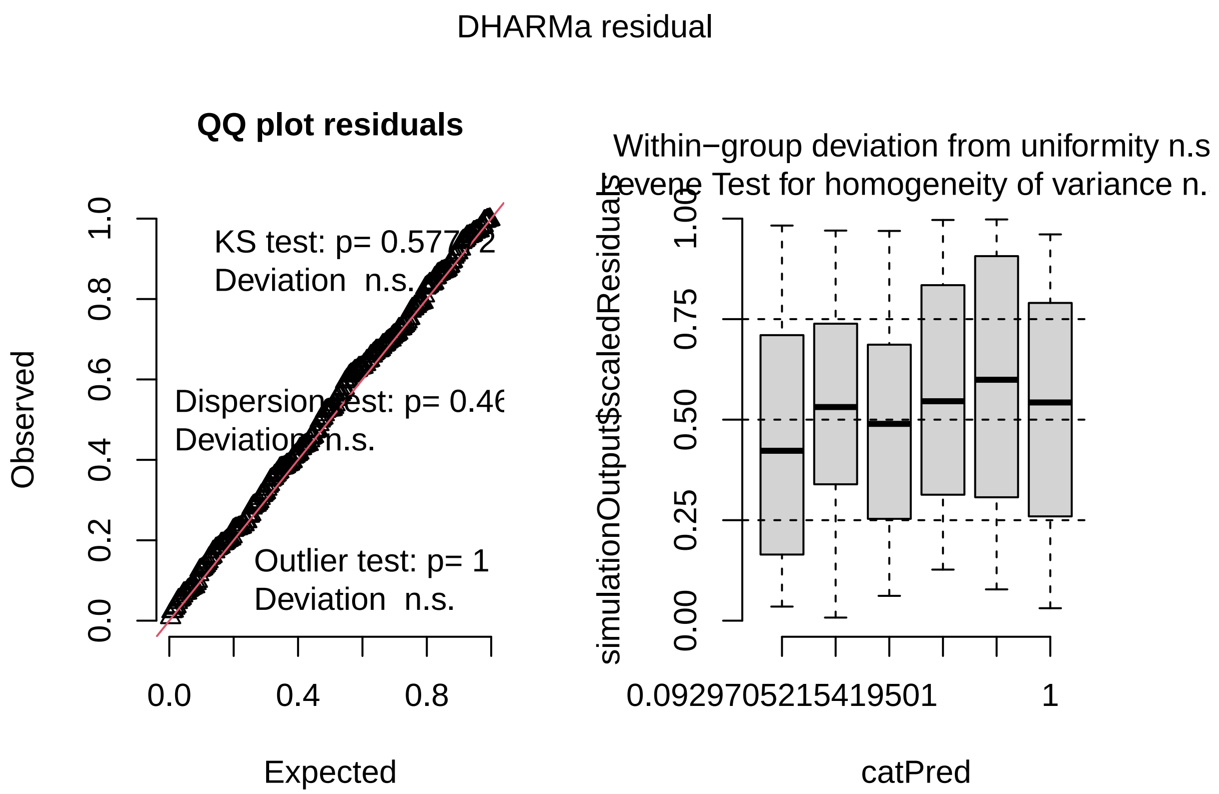

This works. Not ideal, but acceptable.

```
Anova(mc3m)
```

```
## Analysis of Deviance Table (Type II Wald chisquare tests)
##
## Response: choice
##                                  Chisq Df Pr(>Chisq)
## as.factor(Visit_number)         0.4024  2    0.81775
## order                           3.3326  1    0.06792 .
## as.factor(Visit_number):order 0.7512  2    0.68689
## ---
## Signif. codes:  0 '***' 0.001 '**' 0.01 '*' 0.05 '.' 0.1 ' ' 1
```

there is no effect of any variable. will proceed with a post-hoc to see if overall they are different from chance level

```
e <- emmeans(mc3m, ~1, type='response')
t<-test(e, adjust='bonferroni')
t
```

```
##  1         prob    SE  df null t.ratio p.value
##  overall 0.466 0.122 232  0.5  -0.279  0.7803
##
## Results are averaged over the levels of: Visit_number, order
## Tests are performed on the logit scale
```

```
t<-as.data.frame(t)
interpret_cohens_d(t_to_d(t = t$t.ratio, df = t$df))
```

```
## d    |       95% CI | Interpretation
## -------------------------------------
```

```
## 0.04 | [0.29, 0.22] |     very small
##
## - Interpretation rule: cohen1988
```

```
interpret_eta_squared(t_to_eta2(t = t$t.ratio, df = t$df))
```

```
## Eta2 (partial) |       95% CI | Interpretation
## ----------------------------------------------
## 3.36e-04       | [0.00, 1.00] |     very small
##
## - One-sided CIs: upper bound fixed at [1.00].
## - Interpretation rule: field2013
```

there is no significant difference from chance level. There seems to be a slight tendency to prefer the segregated option. I will for completeness look at the different variables.

```
e <- emmeans(mc3m, ~Visit_number*order, type='response')
test(e, adjust='bonferroni')
```

```
##  Visit_number order                    prob    SE  df null t.ratio p.value
##             9 Initial_Choice_Binomial 0.347 0.142 232  0.5  -1.012  1.0000
##            10 Initial_Choice_Binomial 0.430 0.152 232  0.5  -0.454  1.0000
##            11 Initial_Choice_Binomial 0.388 0.148 232  0.5  -0.733  1.0000
##             9 Final_Choice_Binomial   0.603 0.149 232  0.5   0.670  1.0000
##            10 Final_Choice_Binomial   0.560 0.153 232  0.5   0.388  1.0000
##            11 Final_Choice_Binomial   0.473 0.154 232  0.5  -0.174  1.0000
##
## P value adjustment: bonferroni method for 6 tests
## Tests are performed on the logit scale
```

Pheromone analysis

```
cond3PheroLong <- melt(cond3, measure.vars = c("Pherom_back_1", "Pherom_back_2","Pherom_back_3"),
                  value.name = "Pheromone", na.rm = TRUE, variable.name = "section")

mc3p <- glmmTMB(Pheromone~section*Treatment+(1|Visit_number/Colony/Ant_ID_long),
                data=cond3PheroLong, family = poisson())
simres <- simulateResiduals(mc3p)
plot(simres, factor=TRUE)
```

```
## DHARMa:testOutliers with type = binomial may have inflated Type I error rates for integer-valued dis
```

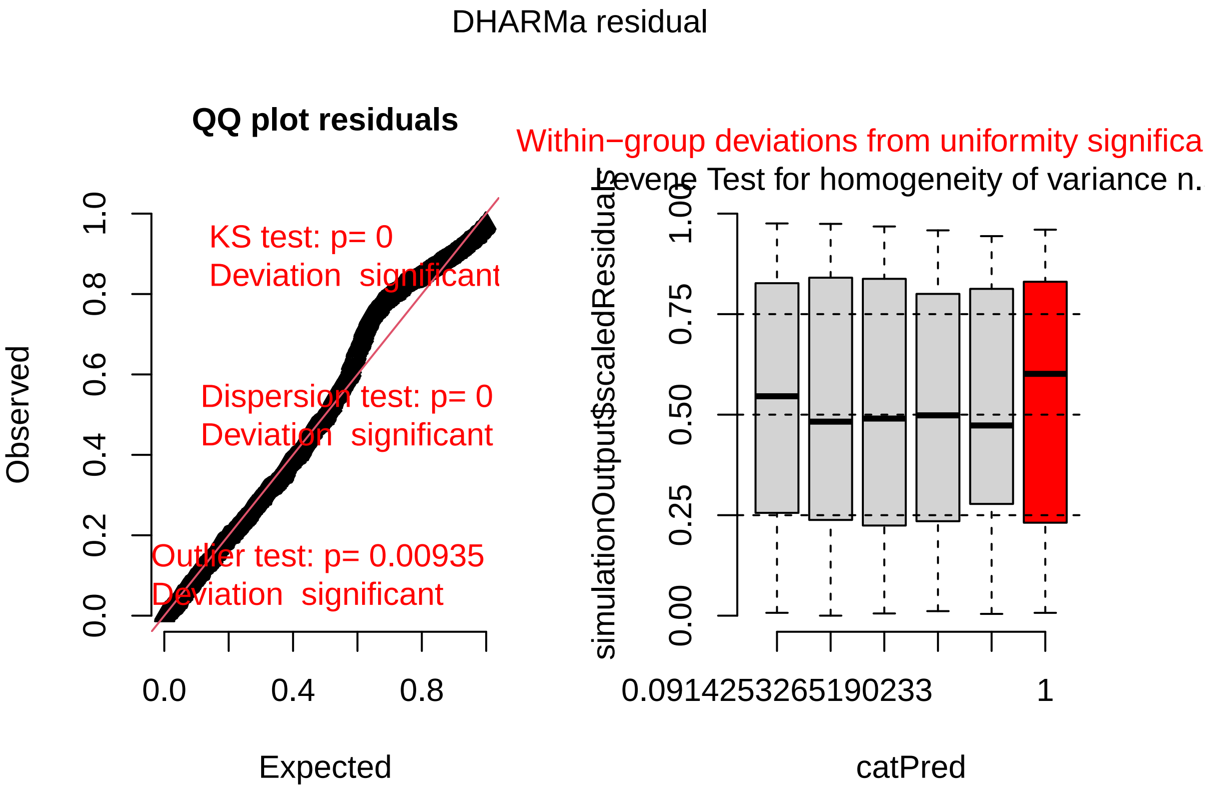

model is not good. probably zero inflated. Will check

```
hist(cond3PheroLong$Pheromone)
```

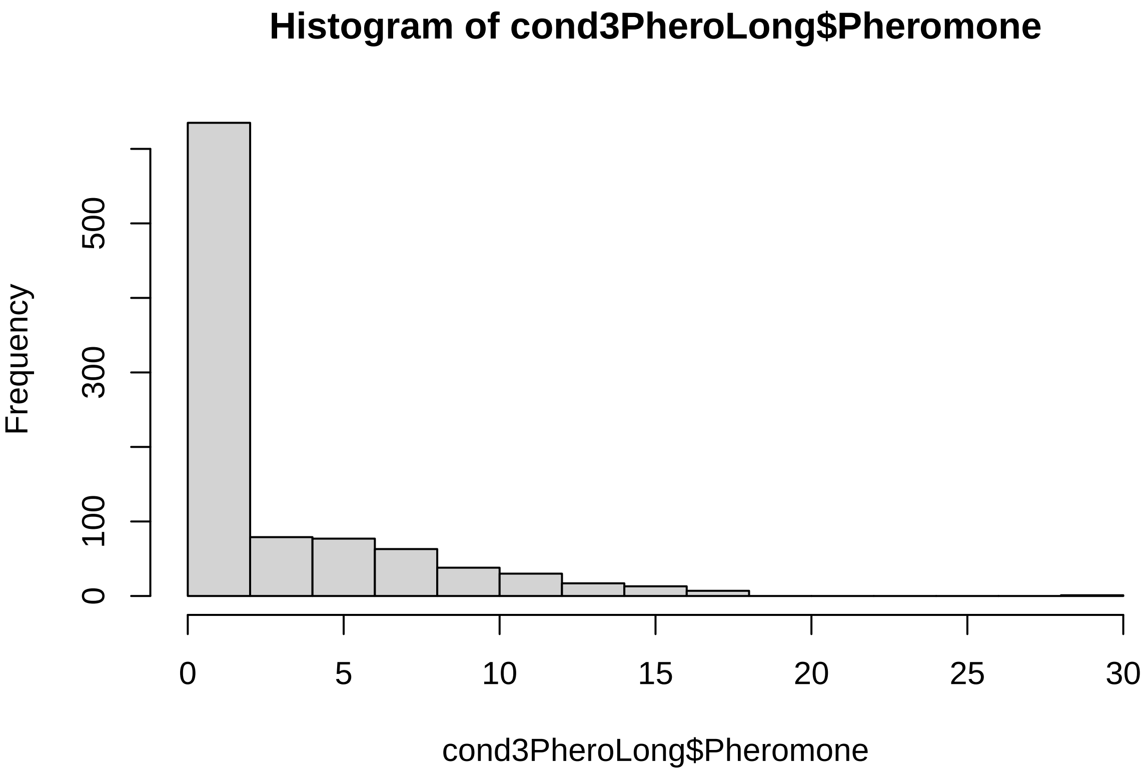

```r
#indeed zero inflated
mc3p <- glmmTMB(Pheromone~section*Treatment+(1|Visit_number/Colony/Ant_ID_long), data=cond3PheroLong, zi
simres <- simulateResiduals(mc3p)
plot(simres, factor=TRUE)
```

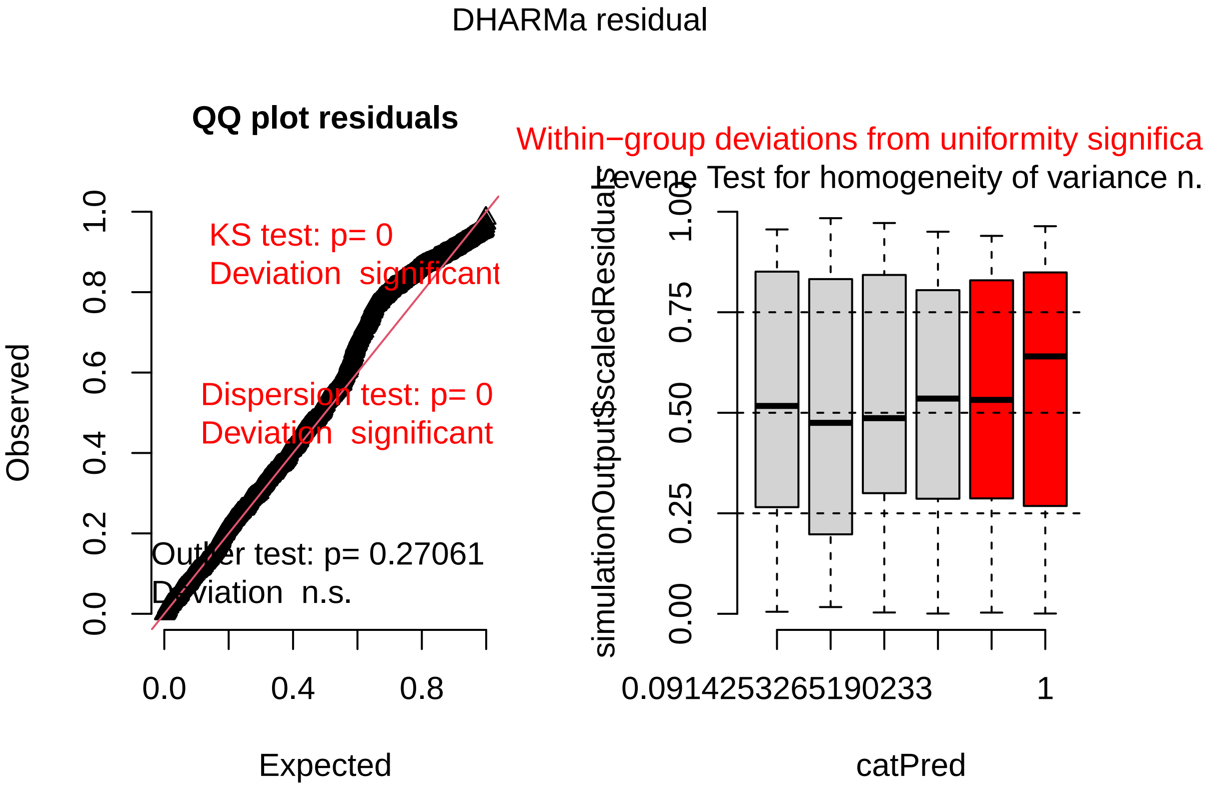

we fixed the low part of the curve, but there is still an inflection to the top. I think it's because there is still a small peak at 30. Does this call for a tweedie?

```r
mc3p <- glmmTMB(Pheromone~section*Treatment+(1|Visit_number/Colony/Ant_ID_long), ziformula = ~1,
                data=cond3PheroLong, family = tweedie(link = 'log'))
simres <- simulateResiduals(mc3p)
plot(simres, factor=TRUE)
```

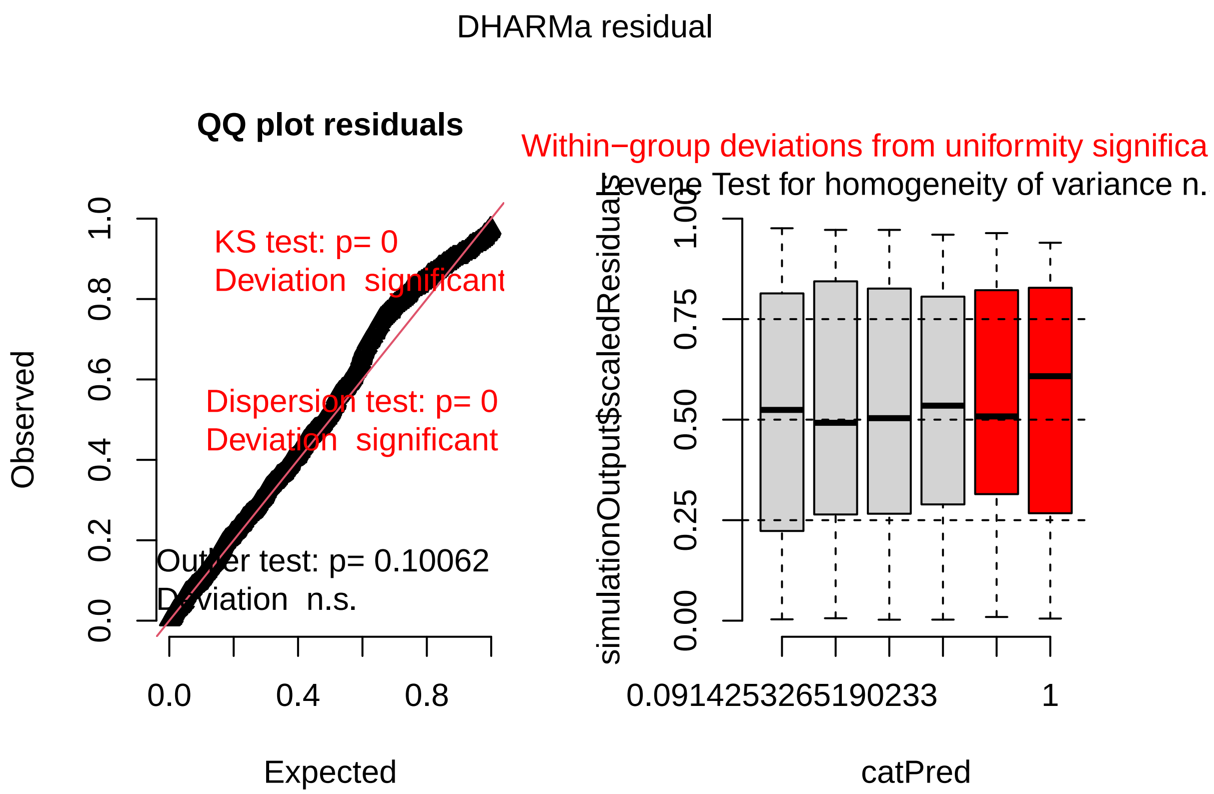

I don't think is getting better than this. will proceed.

```
Anova(mc3p)
```

```
## Analysis of Deviance Table (Type II Wald chisquare tests)
##
## Response: Pheromone
##                       Chisq Df Pr(>Chisq)
## section             24.6063  2  4.537e-06 ***
## Treatment            9.9822  1   0.001581 **
## section:Treatment   13.3183  2   0.001282 **
## ---
## Signif. codes:  0 '***' 0.001 '**' 0.01 '*' 0.05 '.' 0.1 ' ' 1
```

there is in fact an effect of treatment, and it also seems that the pheromone is deposited differently in different sections

```
e <- emmeans(mc3p, ~Treatment*section, type="response")
e
```

```
##  Treatment     section       response    SE  df lower.CL upper.CL
##  All_Segregated Pherom_back_1    0.492 0.114 948    0.312    0.775
##  Bundled        Pherom_back_1    1.315 0.273 948    0.874    1.977
##  All_Segregated Pherom_back_2    0.466 0.108 948    0.295    0.736
##  Bundled        Pherom_back_2    0.857 0.181 948    0.566    1.299
##  All_Segregated Pherom_back_3    0.488 0.113 948    0.310    0.770
##  Bundled        Pherom_back_3    0.773 0.165 948    0.509    1.174
##
## Confidence level used: 0.95
## Intervals are back-transformed from the log scale
```

```
t<-contrast(e, list( "SegregatedAllVsBundled" = c(1/3,-1/3,1/3,-1/3,1/3,-1/3),
                   "Section1VsSection2" = c(0.5,0.5,-0.5,-0.5,0,0),
                   "Section2VsSection3" = c(0,0,0.5,0.5,-0.5,-0.5),
                   "Section1VsSection3" = c(0.5,0.5,0,0,-0.5,-0.5),
                   "SegregatedAllSection1vs2"=c(1,0,-1,0,0,0),
                   "SegregatedAllSection2vs3"=c(0,0,1,0,-1,0),
                   "SegregatedAllSection1vs3"=c(1,0,0,0,-1,0),
                   "BundledSection1vs2"=c(0,1,0,-1,0,0),
                   "BundledSection2vs3"=c(0,0,0,1,0,-1),
                   "BundledSection1vs3"=c(0,1,0,0,0,-1)), adjust = "bonferroni")

t
```

```
##  contrast                 ratio     SE  df null t.ratio p.value
##  SegregatedAllVsBundled   0.505 0.1124 948    1  -3.070  0.0220
##  Section1VsSection2       1.272 0.0948 948    1   3.229  0.0128
##  Section2VsSection3       1.029 0.0802 948    1   0.364  1.0000
##  Section1VsSection3       1.309 0.0980 948    1   3.593  0.0034
##  SegregatedAllSection1vs2 1.055 0.1229 948    1   0.459  1.0000
##  SegregatedAllSection2vs3 0.955 0.1113 948    1  -0.399  1.0000
##  SegregatedAllSection1vs3 1.007 0.1161 948    1   0.060  1.0000
##  BundledSection1vs2       1.534 0.1427 948    1   4.600  <.0001
##  BundledSection2vs3       1.109 0.1149 948    1   0.998  1.0000
##  BundledSection1vs3       1.701 0.1626 948    1   5.557  <.0001
##
## P value adjustment: bonferroni method for 10 tests
## Tests are performed on the log scale
```

```
t<-as.data.frame(t)
interpret_cohens_d(t_to_d(t = t$t.ratio, df = t$df))
```

```
## d        |       95% CI | Interpretation
## ----------------------------------------
## 0.20     | [0.33, 0.07] |     very small
## 0.21     | [0.08, 0.34] |          small
## 0.02     | [0.10, 0.15] |     very small
## 0.23     | [0.11, 0.36] |          small
## 0.03     | [0.10, 0.16] |     very small
## 0.03     | [0.15, 0.10] |     very small
## 3.89e-03 | [0.12, 0.13] |     very small
## 0.30     | [0.17, 0.43] |          small
## 0.06     | [0.06, 0.19] |     very small
## 0.36     | [0.23, 0.49] |          small
##
## - Interpretation rule: cohen1988
```

```
interpret_eta_squared(t_to_eta2(t = t$t.ratio, df = t$df))
```

```
## Eta2 (partial) |       95% CI | Interpretation
## ----------------------------------------------
## 9.85e-03       | [0.00, 1.00] |     very small
## 0.01           | [0.00, 1.00] |          small
## 1.40e-04       | [0.00, 1.00] |     very small
## 0.01           | [0.00, 1.00] |          small
## 2.22e-04       | [0.00, 1.00] |     very small
```

```
## 1.68e-04       | [0.00, 1.00] |     very small
## 3.78e-06       | [0.00, 1.00] |     very small
## 0.02           | [0.01, 1.00] |          small
## 1.05e-03       | [0.00, 1.00] |     very small
## 0.03           | [0.02, 1.00] |          small
##
## - One-sided CIs: upper bound fixed at [1.00].
## - Interpretation rule: field2013
```

```
etoplot <- emmeans(mc3p, ~Treatment, type="response")
```

```
## NOTE: Results may be misleading due to involvement in interactions
```

```
exp3PheroToplot <- as.data.frame(etoplot)
write.csv(exp3PheroToplot,paste0(pathtofile, 'cond3Phero.csv'))
```

The ant deposited overall more pheromone for the bundled over the segregated option. Moreover, in this condition specifically, they deposited more pheromone in section 1 (nearest to the end of runway) in respect to section 2 (middle) or 3 (near the bridge)

Overall pheromone Graph

```
Cond1Phero = pd.DataFrame(r.exp1PheroToplot)
Cond2Phero = pd.DataFrame(r.exp2PheroToplot)
Cond3Phero = pd.DataFrame(r.exp3PheroToplot)
Cond1Phero['Condition'] = 'Condition 1'
Cond2Phero['Condition'] = 'Condition 2'
Cond3Phero['Condition'] = 'Condition 3'

allPhero = pd.concat([Cond1Phero,Cond2Phero,Cond3Phero])

sns.scatterplot(data=allPhero, x='Treatment', y='response', hue='Condition')
plt.vlines(Cond1Phero['Treatment'].values,
          ymin=np.subtract(Cond1Phero['response'].values,Cond1Phero['SE'].values),
          ymax=np.add(Cond1Phero['response'].values,Cond1Phero['SE'].values),
           colors='#1f77b4')
plt.vlines(Cond2Phero['Treatment'].values,
          ymin=np.subtract(Cond2Phero['response'].values,Cond2Phero['SE'].values),
          ymax=np.add(Cond2Phero['response'].values,Cond2Phero['SE'].values),
           colors='#ff7f0e')
plt.vlines(Cond3Phero['Treatment'].values,
          ymin=np.subtract(Cond3Phero['response'].values,Cond3Phero['SE'].values),
          ymax=np.add(Cond3Phero['response'].values,Cond3Phero['SE'].values),
           colors='#2ca02c')
plt.ylim((0,3))
```

```
## (0.0, 3.0)
```

```
plt.show()
```

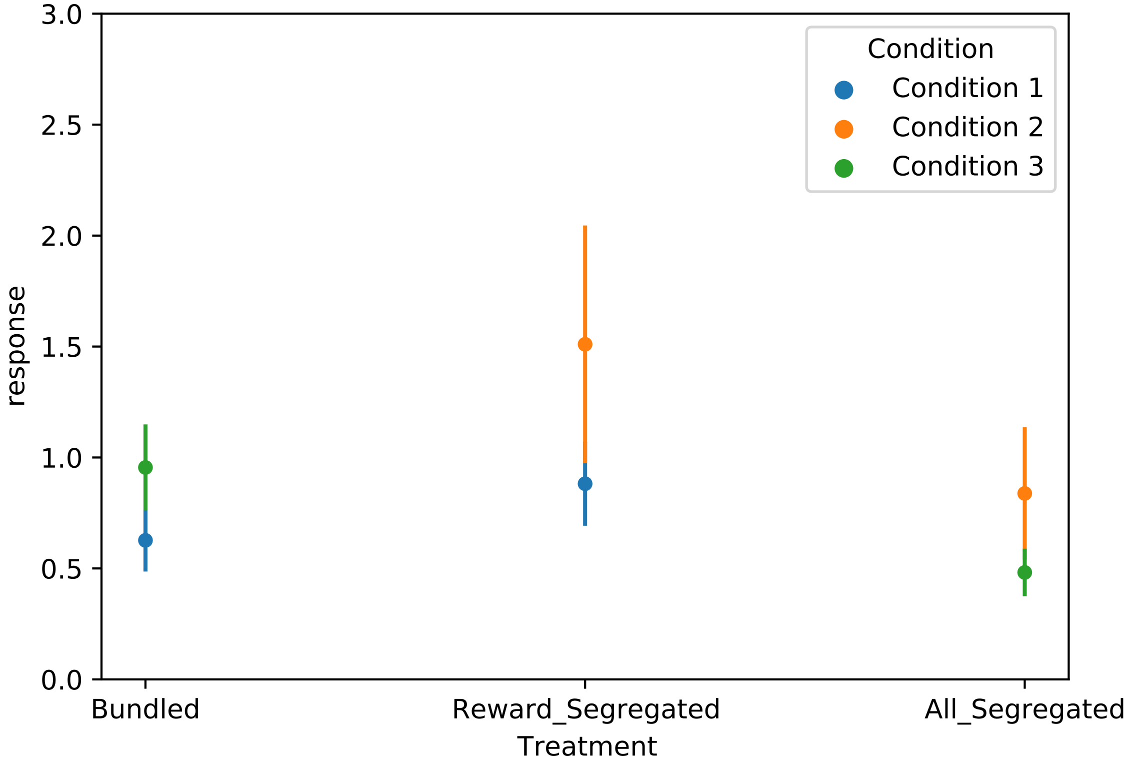

Further investigation

Is the difference between condition caused by contrast effect?

We observed that pheromone deposited is higher for the segregated reward condition over the segregated cost one, and the bundled results higher than the segregated cost. As per our hypothesis, the segregated reward should have been preferred over the bundled, but we do not observe a higher deposition here. A possible hypothesis is that our treatment successfully segregated costs (i.e. distance) but not reward. With no difference between rewards in the conditions, only the costs difference matters. An alternative, non-exclusive hypothesis, may be related to contrast effect. The preference is developed not absolutely, but only in comparison with the other experienced option. To test this hypothesis, we can observe the pheromone deposited in the first visit rather than in the subsequent ones, as in the first visit no contrast is available. Also, in this case, the first experienced treatment will have an effect on the overall preference. Given the fact that we are adding two factors, we are going ignore the three pheromone deposition sections, and only count the total amount.

Condition 1

```
mofc1p <- glmmTMB(Pheromone~Treatment*Visit_number*First_Treatment+(1|Visit_number/Colony/Ant_ID_long),
                  ziformula = ~1, data=cond1PheroLong, family = tweedie(link = 'log'))
simres <- simulateResiduals(mofc1p)

plot(simres, factor=TRUE)
```

```
## Warning in plot.window(...): parametro grafico "factor" non valido
```

```
## Warning in plot.xy(xy, type, ...): parametro grafico "factor" non valido
```

```
## Warning in title(...): parametro grafico "factor" non valido
```

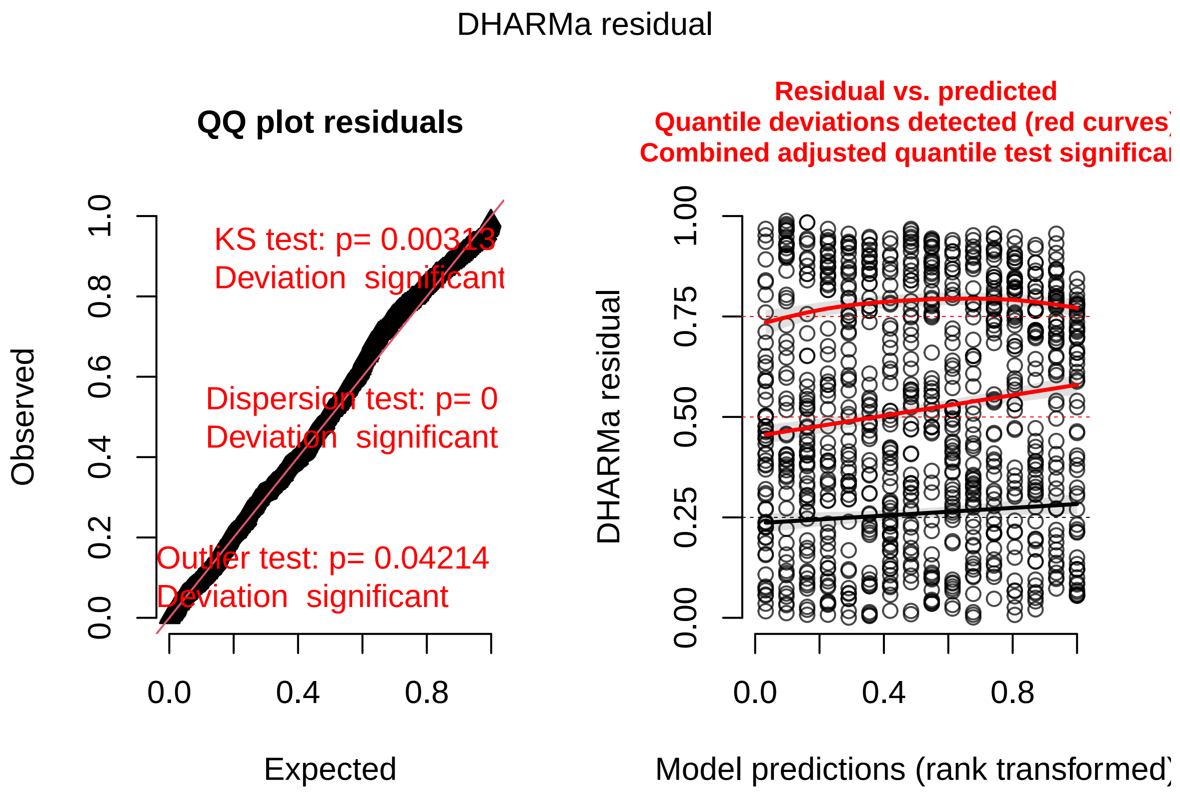


```
Anova(mofc1p)
```

```
## Analysis of Deviance Table (Type II Wald chisquare tests)
##
## Response: Pheromone
##                                      Chisq Df Pr(>Chisq)
## Treatment                           1.3150  1   0.251488
## Visit_number                        1.9214  1   0.165702
## First_Treatment                     7.2418  1   0.007123 **
## Treatment:Visit_number              1.1950  1   0.274325
## Treatment:First_Treatment           0.2852  1   0.593336
## Visit_number:First_Treatment        0.7933  1   0.373102
## Treatment:Visit_number:First_Treatment 2.3334  1   0.126621
## ---
## Signif. codes:  0 '***' 0.001 '**' 0.01 '*' 0.05 '.' 0.1 ' ' 1
```

```
e <- emmeans(mofc1p, ~Treatment*First_Treatment, type='response')
```

```
## NOTE: Results may be misleading due to involvement in interactions
```

```
e
```

```
##  Treatment          First_Treatment    response    SE  df lower.CL upper.CL
##  Bundled            Bundled               0.580 0.171 946    0.326     1.03
##  Reward_Segregated Bundled               0.648 0.191 946    0.363     1.16
##  Bundled            Reward_Segregated     0.984 0.276 946    0.568     1.71
##  Reward_Segregated Reward_Segregated     1.485 0.396 946    0.880     2.51
##
## Confidence level used: 0.95
```

```
## Intervals are back-transformed from the log scale
```

```
contrast(e, list("BundledVsSegregatedReward" = c(0.5,-0.5,0.5,-0.5),
                 "Bundled_FirstVsSegregatedReward_First" = c(0.5,0.5,0-0.5,-0.5)),
         adjust = "bonferroni")
```

```
##  contrast                              ratio    SE  df null t.ratio p.value
##  BundledVsSegregatedReward             0.770 0.185 946    1  -1.089  0.5531
##  Bundled_FirstVsSegregatedReward_First 0.507 0.127 946    1  -2.718  0.0134
##
## P value adjustment: bonferroni method for 2 tests
## Tests are performed on the log scale
```

We observe a difference between the first treatments experienced. Looking at the post-hoc, we see that the effect is driven by the difference between treatments WHEN that treatment is the first encountered. this is almost certainly an effect of the low (almost always 0) pheromone deposition.There may also be a contrast effect, but here we afraid is going to be masked by the first visit. Will proceed for completeness, but we intend testing the results removing visit 1

Condition 2

```
mofc2p <- glmmTMB(Pheromone~Treatment*Visit_number*First_Treatment+(1|Visit_number/Colony/Ant_ID_long),
                  ziformula = ~1, data=cond2PheroLong, family = tweedie(link = 'log'))
simres <- simulateResiduals(mofc2p)

plot(simres, factor=TRUE)
```

```
## Warning in plot.window(...): parametro grafico "factor" non valido

## Warning in plot.xy(xy, type, ...): parametro grafico "factor" non valido

## Warning in title(...): parametro grafico "factor" non valido
```

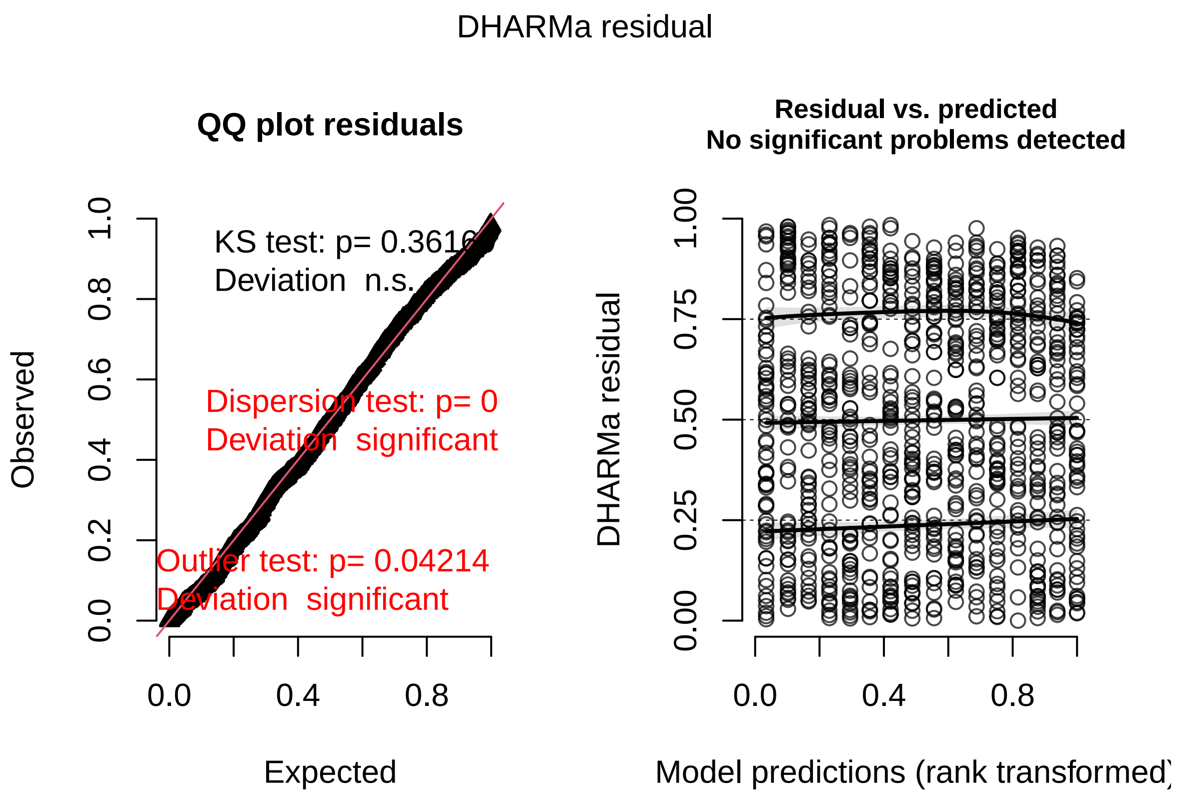


```
Anova(mofc2p)
```

```
## Analysis of Deviance Table (Type II Wald chisquare tests)
##
## Response: Pheromone
##                                        Chisq Df Pr(>Chisq)
## Treatment                            16.2425  1  5.573e-05 ***
## Visit_number                          8.2235  1  0.0041352 **
## First_Treatment                      12.5450  1  0.0003973 ***
## Treatment:Visit_number                0.0166  1  0.8973510
## Treatment:First_Treatment             0.3485  1  0.5549428
## Visit_number:First_Treatment          1.8021  1  0.1794643
## Treatment:Visit_number:First_Treatment  1.2335  1  0.2667238
## ---
## Signif. codes:  0 '***' 0.001 '**' 0.01 '*' 0.05 '.' 0.1 ' ' 1
```

```
e <- emmeans(mofc2p, ~Treatment*First_Treatment, type='response')
```

```
## NOTE: Results may be misleading due to involvement in interactions
```

```
e
```

```
##  Treatment          First_Treatment    response    SE  df lower.CL upper.CL
##  All_Segregated     All_Segregated        0.497 0.138 946    0.288    0.856
##  Reward_Segregated All_Segregated        0.965 0.253 946    0.577    1.614
##  All_Segregated     Reward_Segregated     0.851 0.237 946    0.493    1.469
##  Reward_Segregated Reward_Segregated     2.304 0.596 946    1.387    3.826
##
## Confidence level used: 0.95
## Intervals are back-transformed from the log scale
```

```r
contrast(e, list("SegregatedAllVsSegregatedReward" = c(0.5,-0.5,0.5,-0.5),
                 "SegregatedAll_FirstVsSegregatedReward_First" = c(0.5,0.5,0-0.5,-0.5)), adjust = "bonf
```

```
##  contrast                                    ratio    SE  df null t.ratio
##  SegregatedAllVsSegregatedReward             0.436 0.090 946    1  -4.021
##  SegregatedAll_FirstVsSegregatedReward_First 0.494 0.102 946    1  -3.422
##  p.value
##   0.0001
##   0.0013
##
## P value adjustment: bonferroni method for 2 tests
## Tests are performed on the log scale
```

```r
et <- emtrends(mofc2p, ~Treatment*First_Treatment, var = "Visit_number")
test(et, adjust='bonferroni')
```

```
##  Treatment         First_Treatment   Visit_number.trend    SE  df t.ratio
##  All_Segregated    All_Segregated                0.2230 0.122 946   1.828
##  Reward_Segregated All_Segregated                0.0601 0.111 946   0.542
##  All_Segregated    Reward_Segregated             0.1895 0.118 946   1.600
##  Reward_Segregated Reward_Segregated             0.3297 0.116 946   2.838
##  p.value
##   0.2715
##   1.0000
##   0.4394
##   0.0186
##
## P value adjustment: bonferroni method for 4 tests
```

Very similar story, although distributed in different combinations.

Condition 3

```r
mofc3p <- glmmTMB(Pheromone~Treatment*Visit_number*First_Treatment+(1|Visit_number/Colony/Ant_ID_long),
                  ziformula = ~1, data=cond3PheroLong, family = tweedie(link = 'log'))
simres <- simulateResiduals(mofc3p)

plot(simres, factor=TRUE)
```

```
## Warning in plot.window(...): parametro grafico "factor" non valido
```

```
## Warning in plot.xy(xy, type, ...): parametro grafico "factor" non valido
```

```
## Warning in title(...): parametro grafico "factor" non valido
```

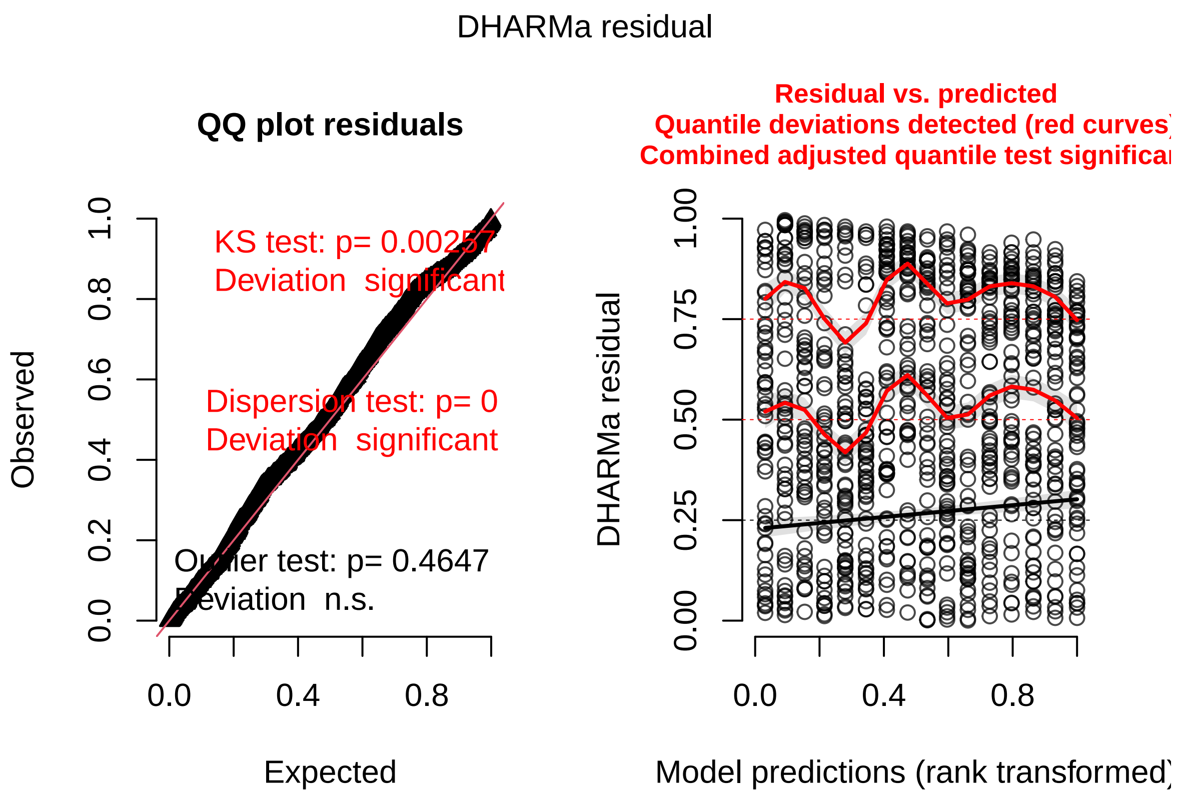


```
Anova(mofc3p)
```

```
## Analysis of Deviance Table (Type II Wald chisquare tests)
##
## Response: Pheromone
##                                       Chisq Df Pr(>Chisq)
## Treatment                            8.3802  1   0.003793 **
## Visit_number                         2.5677  1   0.109069
## First_Treatment                     21.1967  1  4.145e-06 ***
## Treatment:Visit_number               1.8343  1   0.175623
## Treatment:First_Treatment            0.9388  1   0.332576
## Visit_number:First_Treatment         1.9080  1   0.167184
## Treatment:Visit_number:First_Treatment  1.5713  1   0.210025
## ---
## Signif. codes:  0 '***' 0.001 '**' 0.01 '*' 0.05 '.' 0.1 ' ' 1
```

```
e <- emmeans(mofc3p, ~Treatment*First_Treatment, type='response')
```

```
## NOTE: Results may be misleading due to involvement in interactions
```

```
e
```

```
##  Treatment     First_Treatment response     SE  df lower.CL upper.CL
##  All_Segregated All_Segregated     1.018 0.2589 946    0.618    1.677
##  Bundled       All_Segregated     1.520 0.3837 946    0.926    2.494
##  All_Segregated Bundled           0.280 0.0846 946    0.154    0.506
##  Bundled       Bundled           0.719 0.1918 946    0.426    1.214
##
## Confidence level used: 0.95
## Intervals are back-transformed from the log scale
```

```r
contrast(e, list("SegregatedAllVsBundled" = c(0.5,-0.5,0.5,-0.5),
                 "SegregatedAll_FirstVsBundled_First" = c(0.5,0.5,0-0.5,-0.5)), adjust = "bonferroni")
```

```
##  contrast                           ratio    SE  df null t.ratio p.value
##  SegregatedAllVsBundled              0.51 0.112 946    1  -3.074  0.0043
##  SegregatedAll_FirstVsBundled_First  2.77 0.620 946    1   4.563  <.0001
##
## P value adjustment: bonferroni method for 2 tests
## Tests are performed on the log scale
```

```r
et <- emtrends(mofc3p, ~Treatment, var = "Visit_number")
```

```
## NOTE: Results may be misleading due to involvement in interactions
```

```r
test(et, adjust='bonferroni')
```

```
##  Treatment      Visit_number.trend     SE  df t.ratio p.value
##  All_Segregated             0.1585 0.0836 946   1.896  0.1165
##  Bundled                    0.0384 0.0781 946   0.492  1.0000
##
## Results are averaged over the levels of: First_Treatment
## P value adjustment: bonferroni method for 2 tests
```

From the analysis, it appears that depending on the first experienced treatment, the overall pheromone deposition changes. curiously, this doesn't seem to influence preference: the pheromone deposited is overall higher when the "All segregated" treatment is encountered first over the "bundled". This model also confirms the overall higher deposition for the Bundled option. Regarding the progression over visits, only for the All_segregated option we observe an increase in deposition over time. Let's plot to make sense of this.

Graph

```r
ggplot(cond1PheroLong, aes(x=as.factor(Visit_number), y=Pheromone, color=Treatment))+
facet_wrap(~First_Treatment)+
geom_boxplot()+
geom_jitter(width=0.1, height = 0)
```

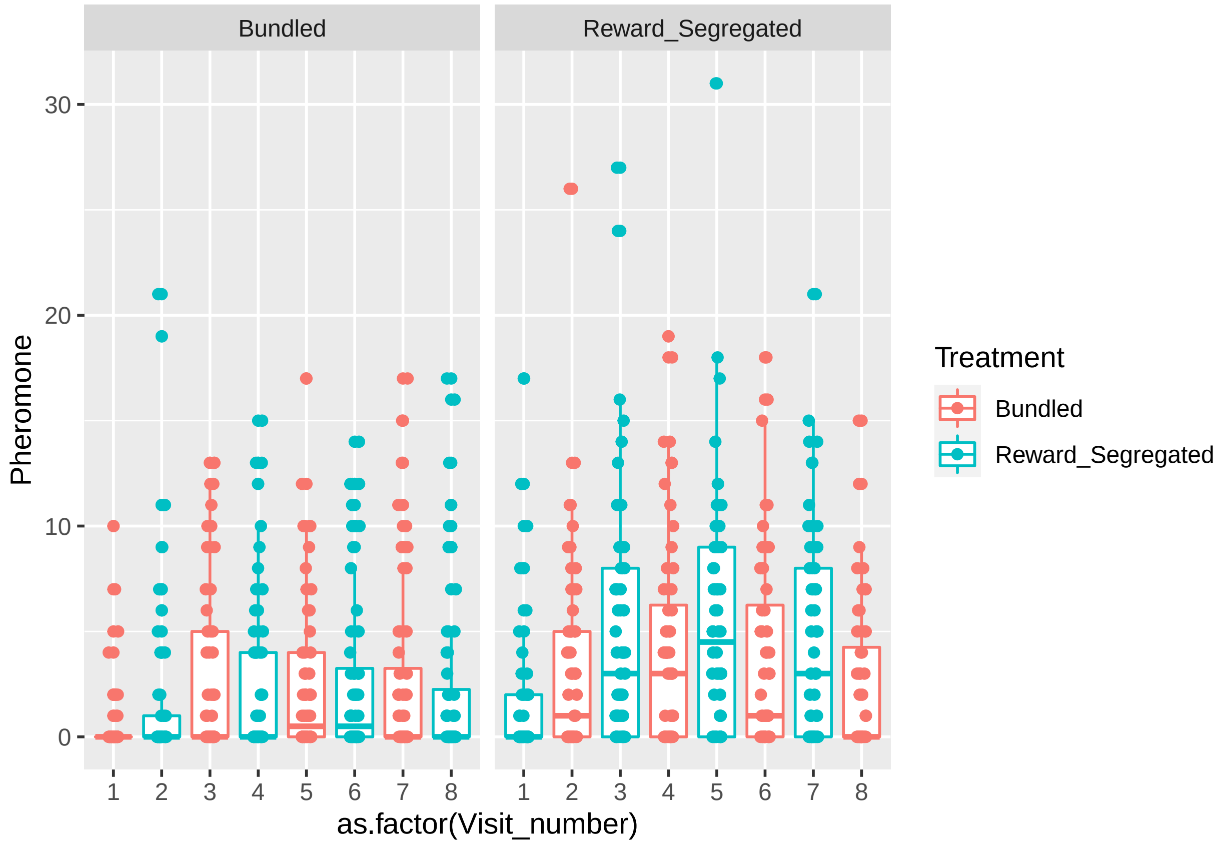


```
ggplot(cond2PheroLong, aes(x=as.factor(Visit_number), y=Pheromone, color=Treatment))+
facet_wrap(~First_Treatment)+
geom_boxplot()+
geom_jitter(width=0.1, height = 0)
```

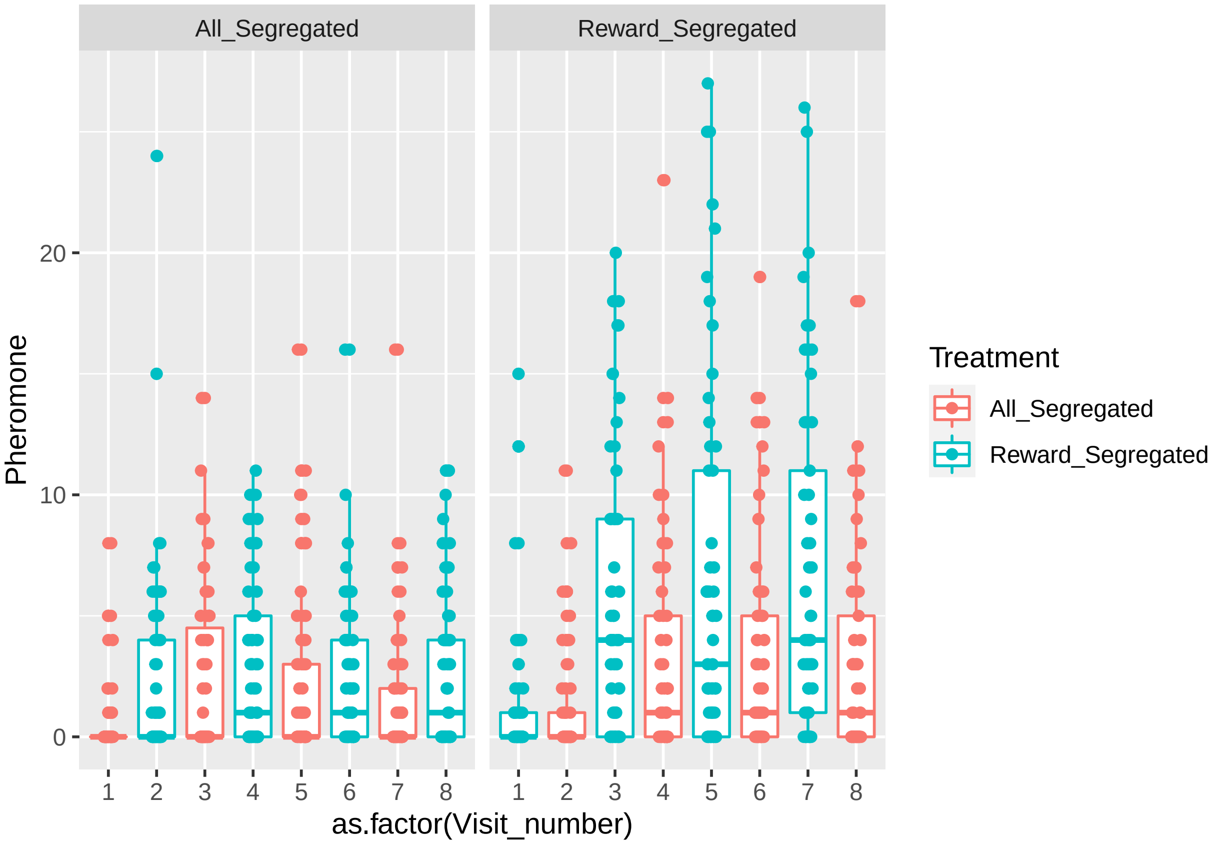


```
ggplot(cond3PheroLong, aes(x=as.factor(Visit_number), y=Pheromone, color=Treatment))+
facet_wrap(~First_Treatment)+
geom_boxplot()+
geom_jitter(width=0.1, height = 0)
```

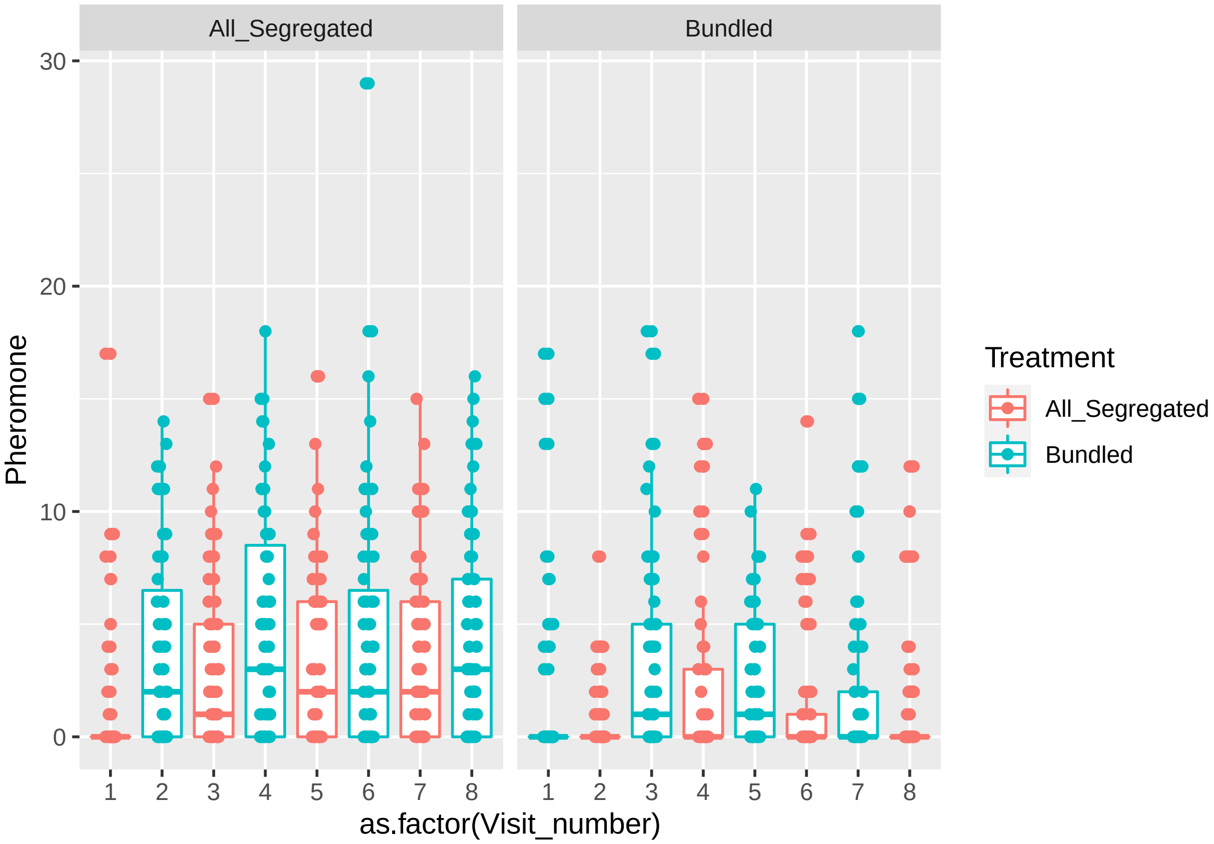

The effects are overall very mild, and instead of helping to explain the main effect they are just adding more questions that we do not have the statistical power to answer to. Rather than start fishing for smaller and smaller effects, we will proceed with a new set of models, dropping the first visit given the big difference with the others, but maintaining the first experienced treatment, as it seems to be able to provide useful insights.

No visit 1

Condition 1

```
cond1PheroLongNoVisit1 <- subset(cond1PheroLong, cond1PheroLong$Visit_number > 1)

mofc1p <- glmmTMB(Pheromone~Treatment*First_Treatment+(1|Visit_number/Colony/Ant_ID_long),
                  ziformula = ~1, data=cond1PheroLongNoVisit1, family = tweedie(link = 'log'))
simres <- simulateResiduals(mofc1p)

plot(simres, factor=TRUE)
```

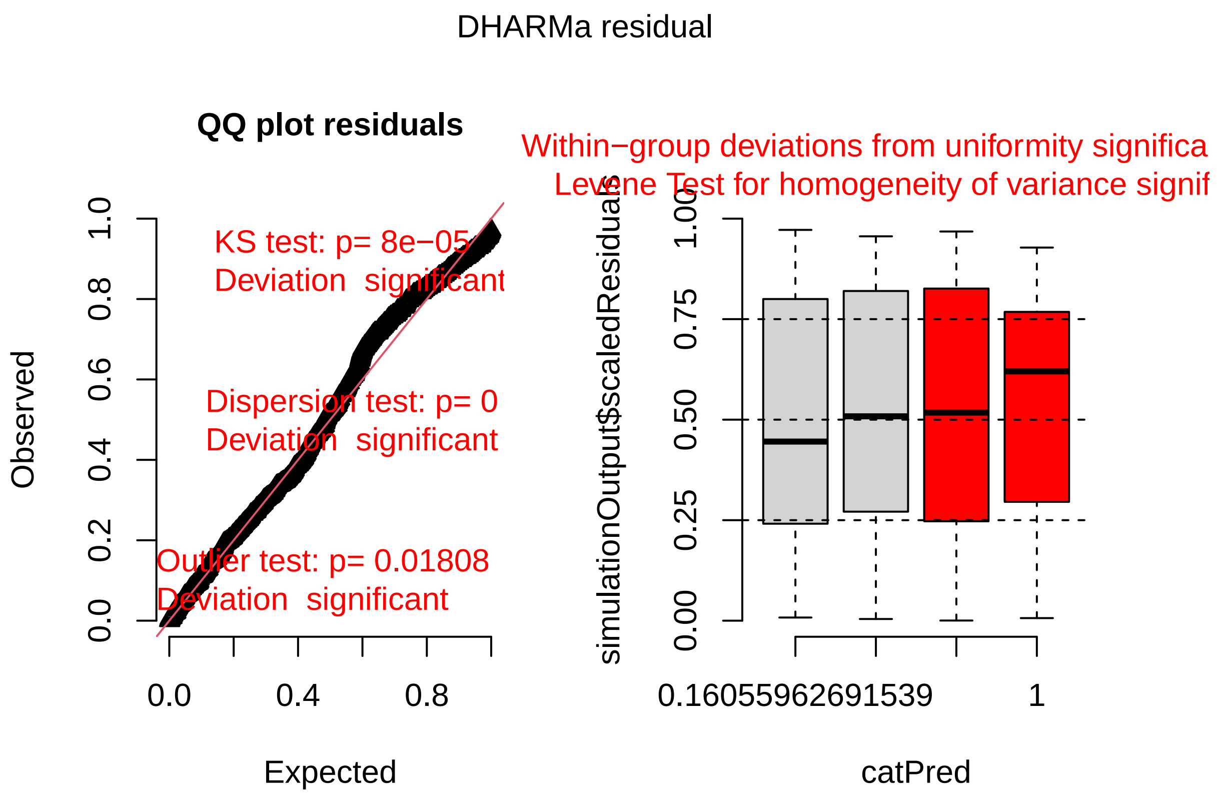


```
Anova(mofc1p)
```

```
## Analysis of Deviance Table (Type II Wald chisquare tests)
##
## Response: Pheromone
##                              Chisq Df Pr(>Chisq)
## Treatment                   1.6959  1    0.19283
## First_Treatment             5.8514  1    0.01556 *
## Treatment:First_Treatment 1.8884  1    0.16939
## ---
## Signif. codes:  0 '***' 0.001 '**' 0.01 '*' 0.05 '.' 0.1 ' ' 1
```

```
e <- emmeans(mofc1p, ~Treatment*First_Treatment, type='response')
e
```

```
##  Treatment          First_Treatment    response    SE  df lower.CL upper.CL
##  Bundled            Bundled               0.779 0.247 830    0.419     1.45
##  Reward_Segregated Bundled               0.689 0.193 830    0.398     1.19
##  Bundled            Reward_Segregated     0.939 0.257 830    0.548     1.61
##  Reward_Segregated Reward_Segregated     1.949 0.568 830    1.100     3.45
##
## Confidence level used: 0.95
## Intervals are back-transformed from the log scale
```

```
contrast(e, list("BundVsRew_seg" = c(0.5,-0.5,0.5,-0.5),
                 "Bund_FirstVsRew_seg_First" = c(0.5,0.5,0-0.5,-0.5),
                 "Bund_whenFirstVswhenSecond"= c(1,0,-1,0),
                 "Rew_seg_whenFirstVswhenSecond"= c(0,1,0,-1),
                 "BundVsRew_seg_whenBundFirst" = c(1,-1,0,0),
                 "BundVsRew_seg_whenRew_segFirst" = c(0,0,1,-1)), adjust = "bonferroni")
```

```
##  contrast                         ratio    SE  df null t.ratio p.value
##  BundVsRew_seg                    0.738 0.181 830    1  -1.239  1.0000
##  Bund_FirstVsRew_seg_First        0.542 0.139 830    1  -2.382  0.1048
##  Bund_whenFirstVswhenSecond       0.830 0.339 830    1  -0.457  1.0000
##  Rew_seg_whenFirstVswhenSecond    0.354 0.141 830    1  -2.613  0.0549
##  BundVsRew_seg_whenBundFirst      1.130 0.457 830    1   0.303  1.0000
##  BundVsRew_seg_whenRew_segFirst   0.482 0.186 830    1  -1.888  0.3561
##
## P value adjustment: bonferroni method for 6 tests
## Tests are performed on the log scale
```

```
forfinalplot1 <- as.data.frame(e)
write.csv(forfinalplot1,paste0(pathtofile, 'Cond1PheroFull.csv'))
```

No significant effect

Condition 2

```
cond2PheroLongNoVisit1 <- subset(cond2PheroLong, cond2PheroLong$Visit_number > 1)

mofc2p <- glmmTMB(Pheromone~Treatment*First_Treatment+(1|Visit_number/Colony/Ant_ID_long),
                  ziformula = ~1, data=cond2PheroLongNoVisit1, family = tweedie(link = 'log'))
simres <- simulateResiduals(mofc2p)

plot(simres, factor=TRUE)
```

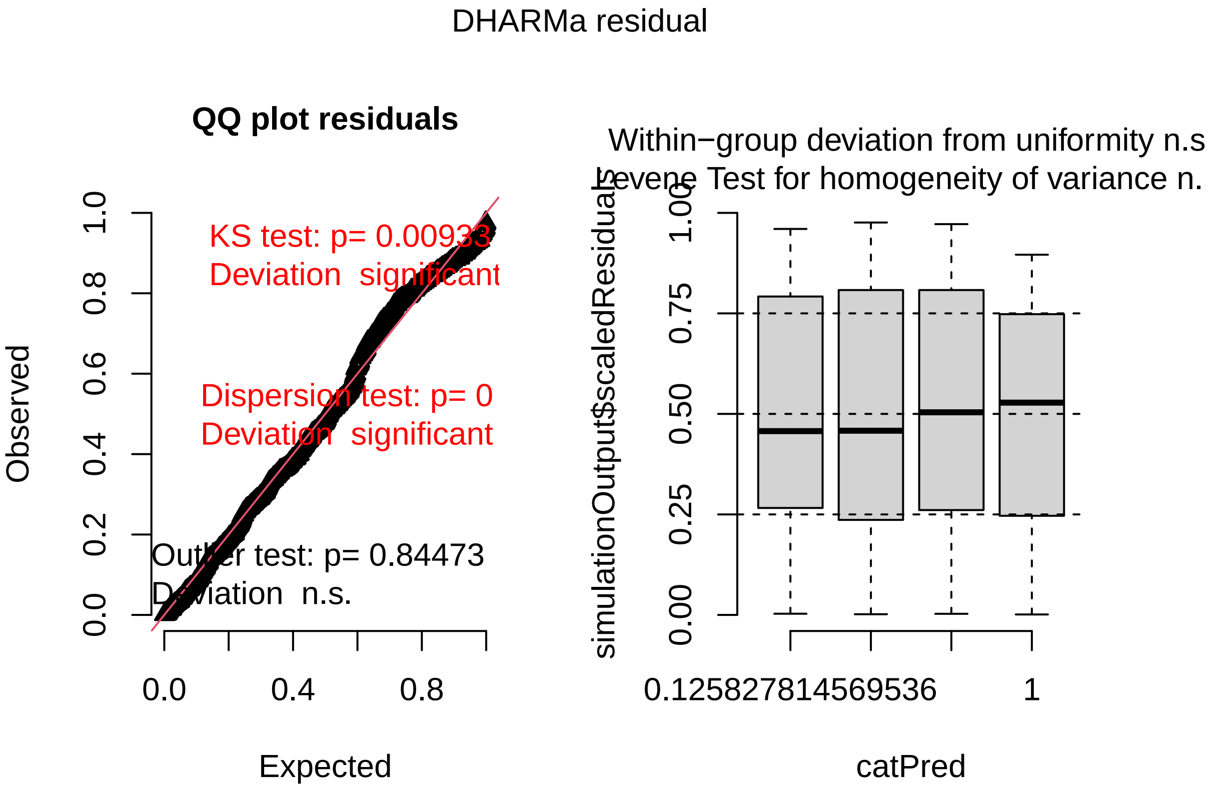


```
Anova(mofc2p)
```

```
## Analysis of Deviance Table (Type II Wald chisquare tests)
##
```

```
## Response: Pheromone
##                              Chisq Df Pr(>Chisq)
## Treatment                  13.8528  1  0.0001977 ***
## First_Treatment            13.1879  1  0.0002818 ***
## Treatment:First_Treatment   2.1383  1  0.1436654
## ---
## Signif. codes:  0 '***' 0.001 '**' 0.01 '*' 0.05 '.' 0.1 ' ' 1
```

```
e <- emmeans(mofc2p, ~Treatment*First_Treatment, type='response')
e
```

```
##  Treatment         First_Treatment   response    SE  df lower.CL upper.CL
##  All_Segregated    All_Segregated       0.700 0.201 830    0.398     1.23
##  Reward_Segregated All_Segregated       0.995 0.241 830    0.619     1.60
##  All_Segregated    Reward_Segregated    0.961 0.244 830    0.584     1.58
##  Reward_Segregated Reward_Segregated    3.250 0.881 830    1.909     5.53
##
## Confidence level used: 0.95
## Intervals are back-transformed from the log scale
```

```
contrast(e, list("All_segVsRew_seg" = c(0.5,-0.5,0.5,-0.5),
                 "All_seg_FirstVsRew_seg_First" = c(0.5,0.5,0-0.5,-0.5),
                 "All_seg_whenFirstVswhenSecond"= c(1,0,-1,0),
                 "Rew_seg_whenFirstVswhenSecond"= c(0,1,0,-1),
                 "All_segVsRew_seg_whenAll_segFirst" = c(1,-1,0,0),
                 "All_segVsRew_seg_whenRew_segFirst" = c(0,0,1,-1)), adjust = "bonferroni")
```

```
##  contrast                          ratio     SE  df null t.ratio p.value
##  All_segVsRew_seg                  0.456 0.0957 830    1  -3.743  0.0012
##  All_seg_FirstVsRew_seg_First      0.472 0.0991 830    1  -3.576  0.0022
##  All_seg_whenFirstVswhenSecond     0.728 0.2690 830    1  -0.859  1.0000
##  Rew_seg_whenFirstVswhenSecond     0.306 0.1092 830    1  -3.318  0.0057
##  All_segVsRew_seg_whenAll_segFirst 0.703 0.2536 830    1  -0.976  1.0000
##  All_segVsRew_seg_whenRew_segFirst 0.296 0.1081 830    1  -3.333  0.0054
##
## P value adjustment: bonferroni method for 6 tests
## Tests are performed on the log scale
```

```
forfinalplot2 <- as.data.frame(e)
write.csv(forfinalplot2,paste0(pathtofile, 'Cond2PheroFull.csv'))
```

Here, the main effect is significant, with a preference for rew_seg. This is mostly evident when rew_seg is encountered first

Condition 3

```
cond3PheroLongNoVisit1 <- subset(cond3PheroLong, cond3PheroLong$Visit_number > 1)

mofc3p <- glmmTMB(Pheromone~Treatment*First_Treatment+(1|Visit_number/Colony/Ant_ID_long),
                  ziformula = ~1, data=cond3PheroLongNoVisit1, family = tweedie(link = 'log'))
simres <- simulateResiduals(mofc3p)

plot(simres, factor=TRUE)
```

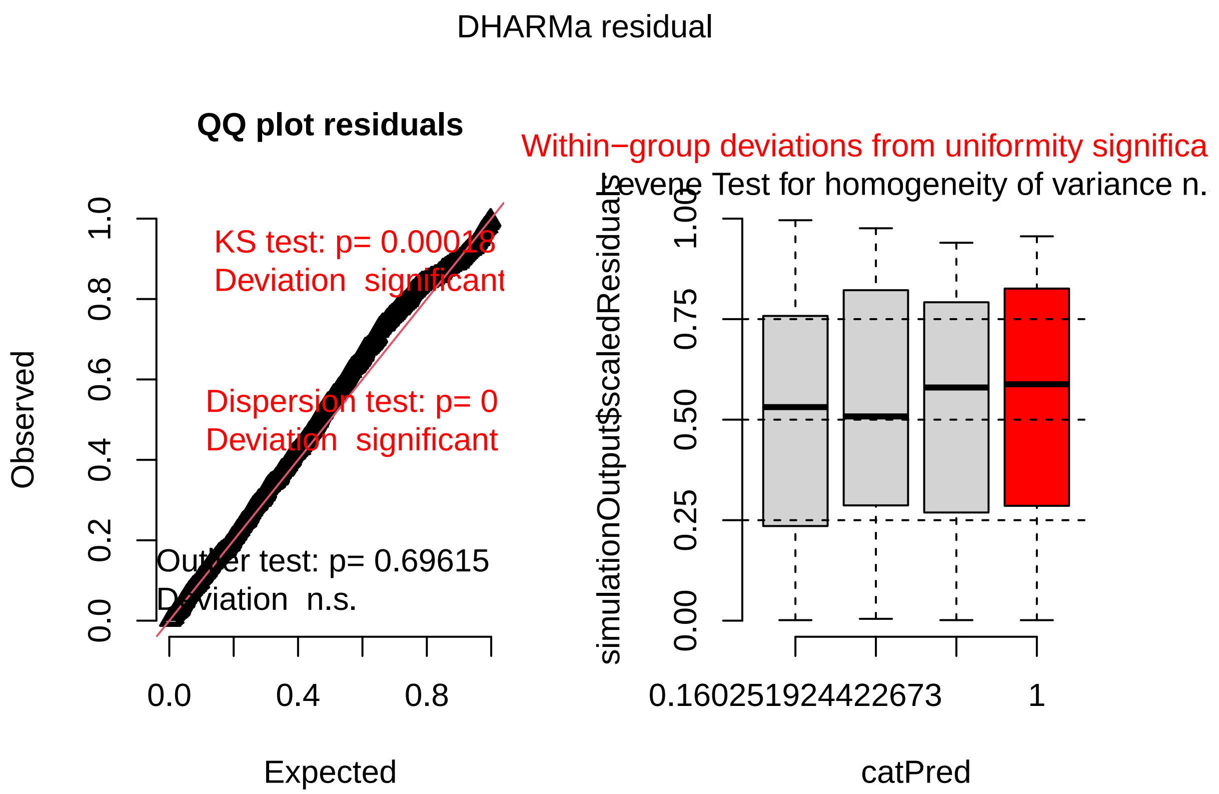


```
Anova(mofc3p)
```

```
## Analysis of Deviance Table (Type II Wald chisquare tests)
##
## Response: Pheromone
##                            Chisq Df Pr(>Chisq)
## Treatment                 6.9127  1   0.008558 **
## First_Treatment          22.6417  1  1.952e-06 ***
## Treatment:First_Treatment  3.4700  1   0.062492 .
## ---
## Signif. codes:  0 '***' 0.001 '**' 0.01 '*' 0.05 '.' 0.1 ' ' 1
```

```
e <- emmeans(mofc3p, ~Treatment*First_Treatment, type='response')
e
```

```
##  Treatment     First_Treatment response     SE  df lower.CL upper.CL
##  All_Segregated All_Segregated    1.485 0.3848 830    0.893    2.470
##  Bundled        All_Segregated    1.621 0.3787 830    1.025    2.564
##  All_Segregated Bundled           0.303 0.0852 830    0.174    0.526
##  Bundled        Bundled           0.940 0.2561 830    0.551    1.605
##
## Confidence level used: 0.95
## Intervals are back-transformed from the log scale
```

```
contrast(e, list("All_SegVsBundeled" = c(0.5,-0.5,0.5,-0.5),
                 "All_Seg_FirstVsBund_First" = c(0.5,0.5,0-0.5,-0.5),
                 "All_Seg_whenFirstVswhenSecond"= c(1,0,-1,0),
                 "Bund_whenFirstVswhenSecond"= c(0,1,0,-1),
                 "All_SegVsBundeled_whenAllSegFirst" = c(1,-1,0,0),
                 "All_SegVsBundeled_whenBundFirst" = c(0,0,1,-1)), adjust = "bonferroni")
```

```
##   contrast                            ratio    SE  df null t.ratio p.value
##   All_SegVsBundeled                   0.543 0.117 830    1  -2.834  0.0283
##   All_Seg_FirstVsBund_First           2.909 0.643 830    1   4.832  <.0001
##   All_Seg_whenFirstVswhenSecond       4.905 1.787 830    1   4.365  0.0001
##   Bund_whenFirstVswhenSecond          1.725 0.603 830    1   1.559  0.7169
##   All_SegVsBundeled_whenAllSegFirst 0.916 0.305 830    1  -0.263  1.0000
##   All_SegVsBundeled_whenBundFirst     0.322 0.120 830    1  -3.032  0.0150
##
## P value adjustment: bonferroni method for 6 tests
## Tests are performed on the log scale
```

```
forfinalplot3 <- as.data.frame(e)
write.csv(forfinalplot3,paste0(pathtofile, 'Cond3PheroFull.csv'))
```

The main effect of treatment remains. Moreover, ants seem to overall deposit more pheromone when they encounter the All_seg option first over the bund. This difference seems to be driven by pheromone deposited in the All_seg option, as pheromone deposited for the bundled is already at bottom leve.

Graph

```
Cond1PheroFinal = pd.DataFrame(r.forfinalplot1)
Cond2PheroFinal = pd.DataFrame(r.forfinalplot2)
Cond3PheroFinal = pd.DataFrame(r.forfinalplot3)

Cond1PheroFinal['Condition'] = 'Condition 1'
Cond2PheroFinal['Condition'] = 'Condition 2'
Cond3PheroFinal['Condition'] = 'Condition 3'

Cond1PheroFinalFirstRewSeg = Cond1PheroFinal[Cond1PheroFinal['First_Treatment'] == "Reward_Segregated"]
Cond1PheroFinalFirstBund = Cond1PheroFinal[Cond1PheroFinal['First_Treatment'] == "Bundled"]

Cond2PheroFinalFirstAllSeg = Cond2PheroFinal[Cond2PheroFinal['First_Treatment'] == "All_Segregated"]
Cond2PheroFinalFirstRewSeg = Cond2PheroFinal[Cond2PheroFinal['First_Treatment'] == "Reward_Segregated"]

Cond3PheroFinalFirstAllSeg = Cond3PheroFinal[Cond3PheroFinal['First_Treatment'] == "All_Segregated"]
Cond3PheroFinalFirstBund = Cond3PheroFinal[Cond3PheroFinal['First_Treatment'] == "Bundled"]

fig, axs = plt.subplots(1,3)

axs[0].scatter(Cond1PheroFinalFirstRewSeg['Treatment'].values,
               Cond1PheroFinalFirstRewSeg['response'].values, c='#1f77b4')
axs[0].vlines(Cond1PheroFinalFirstRewSeg['Treatment'].values,
          ymin=np.subtract(Cond1PheroFinalFirstRewSeg['response'].values,
                           Cond1PheroFinalFirstRewSeg['SE'].values),
          ymax=np.add(Cond1PheroFinalFirstRewSeg['response'].values,
                      Cond1PheroFinalFirstRewSeg['SE'].values),
           colors='#1f77b4')

axs[0].scatter(Cond1PheroFinalFirstBund['Treatment'].values,
               Cond1PheroFinalFirstBund['response'].values, c='#1faab4')
axs[0].vlines(Cond1PheroFinalFirstBund['Treatment'].values,
          ymin=np.subtract(Cond1PheroFinalFirstBund['response'].values,
                           Cond1PheroFinalFirstBund['SE'].values),
          ymax=np.add(Cond1PheroFinalFirstBund['response'].values,
```

```
                      Cond1PheroFinalFirstBund['SE'].values),
           colors='#1faab4')


axs[1].scatter(Cond2PheroFinalFirstRewSeg['Treatment'].values,
               Cond2PheroFinalFirstRewSeg['response'].values, c='#ff7f0e')
axs[1].vlines(Cond2PheroFinalFirstRewSeg['Treatment'].values,
          ymin=np.subtract(Cond2PheroFinalFirstRewSeg['response'].values,
                           Cond2PheroFinalFirstRewSeg['SE'].values),
          ymax=np.add(Cond2PheroFinalFirstRewSeg['response'].values,
                      Cond2PheroFinalFirstRewSeg['SE'].values),
           colors='#ff7f0e')

axs[1].scatter(Cond2PheroFinalFirstAllSeg['Treatment'].values,
               Cond2PheroFinalFirstAllSeg['response'].values, c='#ffaf0e')
axs[1].vlines(Cond2PheroFinalFirstAllSeg['Treatment'].values,
          ymin=np.subtract(Cond2PheroFinalFirstAllSeg['response'].values,
                           Cond2PheroFinalFirstAllSeg['SE'].values),
          ymax=np.add(Cond2PheroFinalFirstAllSeg['response'].values,
                      Cond2PheroFinalFirstAllSeg['SE'].values),
           colors='#ffaf0e')


axs[2].scatter(Cond3PheroFinalFirstAllSeg['Treatment'].values,
               Cond3PheroFinalFirstAllSeg['response'].values, c='#2ca02c')
axs[2].vlines(Cond3PheroFinalFirstAllSeg['Treatment'].values,
          ymin=np.subtract(Cond3PheroFinalFirstAllSeg['response'].values,
                           Cond3PheroFinalFirstAllSeg['SE'].values),
          ymax=np.add(Cond3PheroFinalFirstAllSeg['response'].values,
                      Cond3PheroFinalFirstAllSeg['SE'].values),
           colors='#2ca02c')

axs[2].scatter(Cond3PheroFinalFirstBund['Treatment'].values,
               Cond3PheroFinalFirstBund['response'].values, c='#2c502c')
axs[2].vlines(Cond3PheroFinalFirstBund['Treatment'].values,
          ymin=np.subtract(Cond3PheroFinalFirstBund['response'].values,
                           Cond3PheroFinalFirstBund['SE'].values),
          ymax=np.add(Cond3PheroFinalFirstBund['response'].values,
                      Cond3PheroFinalFirstBund['SE'].values),
           colors='#2c502c')

axs[0].set_ylim((0,5.3))
```

```
## (0.0, 5.3)
```

```
axs[1].set_ylim((0,5.3))
```

```
## (0.0, 5.3)
```

```
axs[2].set_ylim((0,5.3))
```

```
## (0.0, 5.3)
```

```
plt.show()
```

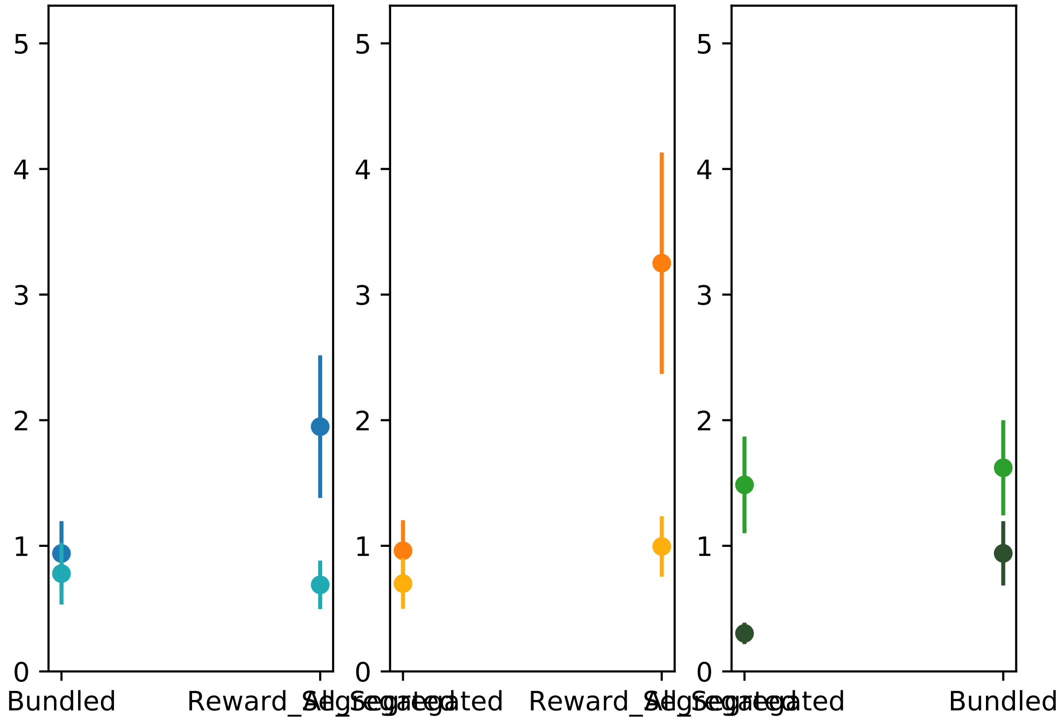
0
1
2
3
4
5
Bundled
Bundled
